# Supplementary material for: Maternal dietary practices, dietary diversity, and nutrient composition of diets of lactating mothers in Jimma Zone, Southwest Ethiopia
Source: PLoS One. 2021 Jul 12;16(7):e0254259. doi: 10.1371/journal.pone.0254259 (PMC8274933; doi:10.1371/journal.pone.0254259)
Supplement: S1 Data — (DOCX) [file pone.0254259.s002.docx]

FREQUENCIES VARIABLES=FOOCO1C FOOCO1D

/ORDER=ANALYSIS.

**Frequencies**

| **Notes** | | |
| --- | --- | --- |
| Output Created | | 26-MAR-2021 10:42:10 |
| Comments | |  |
| Input | Data | C:\Users\PC\Desktop\Maternal dietary practice dataset.sav |
|  | Active Dataset | DataSet1 |
|  | File Label | data file |
|  | Filter | <none> |
|  | Weight | <none> |
|  | Split File | <none> |
|  | N of Rows in Working Data File | 558 |
| Missing Value Handling | Definition of Missing | User-defined missing values are treated as missing. |
|  | Cases Used | Statistics are based on all cases with valid data. |
| Syntax | | FREQUENCIES VARIABLES=FOOCO1C FOOCO1D  /ORDER=ANALYSIS. |
| Resources | Processor Time | 00:00:00.02 |
|  | Elapsed Time | 00:00:00.03 |

[DataSet1] C:\Users\PC\Desktop\Maternal dietary practice dataset.sav

| **Statistics** | | | |
| --- | --- | --- | --- |
|  | | type of stew | Ingredients of stew |
| N | Valid | 558 | 558 |
|  | Missing | 0 | 0 |

**Frequency Table**

| **type of stew** | | | | | |
| --- | --- | --- | --- | --- | --- |
|  | | Frequency | Percent | Valid Percent | Cumulative Percent |
| Valid |  | 236 | 42.3 | 42.3 | 42.3 |
|  | bean | 63 | 11.3 | 11.3 | 53.6 |
|  | bean,cabbage | 4 | .7 | .7 | 54.3 |
|  | bean,lentil | 1 | .2 | .2 | 54.5 |
|  | bean,potato | 12 | 2.2 | 2.2 | 56.6 |
|  | bean,potato,tomato | 1 | .2 | .2 | 56.8 |
|  | bean,tomato | 1 | .2 | .2 | 57.0 |
|  | bean(shiro) | 15 | 2.7 | 2.7 | 59.7 |
|  | bean(shiro),kale | 1 | .2 | .2 | 59.9 |
|  | bean(shiro),potato | 5 | .9 | .9 | 60.8 |
|  | kale | 1 | .2 | .2 | 60.9 |
|  | lentil | 81 | 14.5 | 14.5 | 75.4 |
|  | lentil,bean | 27 | 4.8 | 4.8 | 80.3 |
|  | lentil,bean,cabagge | 1 | .2 | .2 | 80.5 |
|  | lentil,bean,potato | 13 | 2.3 | 2.3 | 82.8 |
|  | lentil,bean(shiro) | 1 | .2 | .2 | 83.0 |
|  | lentil,bean(shiro),potato | 4 | .7 | .7 | 83.7 |
|  | lentil,bean(shiro),potato,kele,rice,tomato | 1 | .2 | .2 | 83.9 |
|  | lentil,bean(shiro),potato,vegetable | 1 | .2 | .2 | 84.1 |
|  | lentil,kale | 1 | .2 | .2 | 84.2 |
|  | lentil,meat | 1 | .2 | .2 | 84.4 |
|  | lentil,potato | 44 | 7.9 | 7.9 | 92.3 |
|  | lentil,potato,kale | 1 | .2 | .2 | 92.5 |
|  | lentil,potato,meat | 1 | .2 | .2 | 92.7 |
|  | lentil,rice | 1 | .2 | .2 | 92.8 |
|  | lentil,vegetable | 1 | .2 | .2 | 93.0 |
|  | lentile,bean(shiro) | 1 | .2 | .2 | 93.2 |
|  | lentile,potato | 4 | .7 | .7 | 93.9 |
|  | letil,potato | 2 | .4 | .4 | 94.3 |
|  | meat | 1 | .2 | .2 | 94.4 |
|  | meat,egg | 1 | .2 | .2 | 94.6 |
|  | potato | 22 | 3.9 | 3.9 | 98.6 |
|  | potato,cabbage | 1 | .2 | .2 | 98.7 |
|  | potato,kale | 1 | .2 | .2 | 98.9 |
|  | potato,pumpkin | 1 | .2 | .2 | 99.1 |
|  | potato,tomato | 2 | .4 | .4 | 99.5 |
|  | potato,vegetable | 1 | .2 | .2 | 99.6 |
|  | tomato | 2 | .4 | .4 | 100.0 |
|  | Total | 558 | 100.0 | 100.0 |  |

| **Ingredients of stew** | | | | | |
| --- | --- | --- | --- | --- | --- |
|  | | Frequency | Percent | Valid Percent | Cumulative Percent |
| Valid |  | 236 | 42.3 | 42.3 | 42.3 |
|  | oil,onion,pepper,tomato,salt | 1 | .2 | .2 | 42.5 |
|  | oil,salt | 2 | .4 | .4 | 42.8 |
|  | onion, oil, peper, salt | 1 | .2 | .2 | 43.0 |
|  | onion,oil,pepper,salt | 183 | 32.8 | 32.8 | 75.8 |
|  | onion,oil,pepper,salt,butter | 4 | .7 | .7 | 76.5 |
|  | onion,oil,pepper,salt,garic | 1 | .2 | .2 | 76.7 |
|  | onion,oil,pepper,salt,garlic | 30 | 5.4 | 5.4 | 82.1 |
|  | onion,oil,pepper,salt,garlic, ginger,spice | 1 | .2 | .2 | 82.3 |
|  | onion,oil,pepper,salt,garlic,ginger | 12 | 2.2 | 2.2 | 84.4 |
|  | onion,oil,pepper,salt,garlic,ginger,spice | 2 | .4 | .4 | 84.8 |
|  | onion,oil,pepper,salt,garlic,green pepper | 2 | .4 | .4 | 85.1 |
|  | onion,oil,pepper,salt,garlic,spice | 5 | .9 | .9 | 86.0 |
|  | onion,oil,pepper,salt,ginger | 3 | .5 | .5 | 86.6 |
|  | onion,oil,pepper,salt,ginger,spice | 1 | .2 | .2 | 86.7 |
|  | onion,oil,pepper,salt,green pepper | 1 | .2 | .2 | 86.9 |
|  | onion,oil,pepper,salt,green pepper,garic | 1 | .2 | .2 | 87.1 |
|  | onion,oil,pepper,salt,green pepper,spice | 1 | .2 | .2 | 87.3 |
|  | onion,oil,pepper,salt,lemon,green pepper | 1 | .2 | .2 | 87.5 |
|  | onion,oil,pepper,salt,spice | 40 | 7.2 | 7.2 | 94.6 |
|  | onion,oil,pepper,salt,spice,spice | 1 | .2 | .2 | 94.8 |
|  | onion,oil,pepper,salt,tomato | 16 | 2.9 | 2.9 | 97.7 |
|  | onion,oil,pepper,salt,tomato,butter | 2 | .4 | .4 | 98.0 |
|  | onion,oil,pepper,salt,tomato,garlic | 3 | .5 | .5 | 98.6 |
|  | onion,oil,pepper,salt,tomato,garlic,butter | 1 | .2 | .2 | 98.7 |
|  | onion,oil,pepper,salt,tomato,garlic,spice | 3 | .5 | .5 | 99.3 |
|  | onion,oil,pepper,salt,tomato,ginger,garlic | 1 | .2 | .2 | 99.5 |
|  | onion,oil,pepper,salt,tomato,spice | 1 | .2 | .2 | 99.6 |
|  | onion,oil,salt | 1 | .2 | .2 | 99.8 |
|  | onion,tomato,oil,salt | 1 | .2 | .2 | 100.0 |
|  | Total | 558 | 100.0 | 100.0 |  |

FREQUENCIES VARIABLES=FOOCO2C FOOCO2D FOOCO3C FOOCO3D FOOCO4C FOOCO4D FOOCO5C FOOCO5D

/ORDER=ANALYSIS.

**Frequencies**

| **Notes** | | |
| --- | --- | --- |
| Output Created | | 26-MAR-2021 10:45:07 |
| Comments | |  |
| Input | Data | C:\Users\PC\Desktop\Maternal dietary practice dataset.sav |
|  | Active Dataset | DataSet1 |
|  | File Label | data file |
|  | Filter | <none> |
|  | Weight | <none> |
|  | Split File | <none> |
|  | N of Rows in Working Data File | 558 |
| Missing Value Handling | Definition of Missing | User-defined missing values are treated as missing. |
|  | Cases Used | Statistics are based on all cases with valid data. |
| Syntax | | FREQUENCIES VARIABLES=FOOCO2C FOOCO2D FOOCO3C FOOCO3D FOOCO4C FOOCO4D FOOCO5C FOOCO5D  /ORDER=ANALYSIS. |
| Resources | Processor Time | 00:00:00.02 |
|  | Elapsed Time | 00:00:00.02 |

[DataSet1] C:\Users\PC\Desktop\Maternal dietary practice dataset.sav

| **Statistics** | | | | | | | | | |
| --- | --- | --- | --- | --- | --- | --- | --- | --- | --- |
|  | | type of stew | Ingredients of stew | type of stew | Ingredients of stew | type of stew | Ingredients of stew | type of stew | Ingredients of stew |
| N | Valid | 558 | 558 | 558 | 558 | 558 | 558 | 558 | 558 |
|  | Missing | 0 | 0 | 0 | 0 | 0 | 0 | 0 | 0 |

**Frequency Table**

| **type of stew** | | | | | |
| --- | --- | --- | --- | --- | --- |
|  | | Frequency | Percent | Valid Percent | Cumulative Percent |
| Valid |  | 328 | 58.8 | 58.8 | 58.8 |
|  | bean | 2 | .4 | .4 | 59.1 |
|  | bean,lentil | 1 | .2 | .2 | 59.3 |
|  | bean,potato | 1 | .2 | .2 | 59.5 |
|  | cabbage | 2 | .4 | .4 | 59.9 |
|  | kale | 207 | 37.1 | 37.1 | 97.0 |
|  | kale,bean | 5 | .9 | .9 | 97.8 |
|  | kale,potato | 7 | 1.3 | 1.3 | 99.1 |
|  | lentil | 2 | .4 | .4 | 99.5 |
|  | potato | 3 | .5 | .5 | 100.0 |
|  | Total | 558 | 100.0 | 100.0 |  |

| **Ingredients of stew** | | | | | |
| --- | --- | --- | --- | --- | --- |
|  | | Frequency | Percent | Valid Percent | Cumulative Percent |
| Valid |  | 329 | 59.0 | 59.0 | 59.0 |
|  | oi,salt | 1 | .2 | .2 | 59.1 |
|  | oil, salt | 3 | .5 | .5 | 59.7 |
|  | oil,salt | 169 | 30.3 | 30.3 | 90.0 |
|  | oil,salt,green pepper | 7 | 1.3 | 1.3 | 91.2 |
|  | onion,oil,pepper,salt | 10 | 1.8 | 1.8 | 93.0 |
|  | onion,oil,salt | 15 | 2.7 | 2.7 | 95.7 |
|  | onion,oil,salt,garlic | 2 | .4 | .4 | 96.1 |
|  | onion,oil,salt,green pepper | 19 | 3.4 | 3.4 | 99.5 |
|  | onion,oil,salt,green pepper,ginger,garlic | 2 | .4 | .4 | 99.8 |
|  | onion,oil,salt,pepper,garlic | 1 | .2 | .2 | 100.0 |
|  | Total | 558 | 100.0 | 100.0 |  |

| **type of stew** | | | | | |
| --- | --- | --- | --- | --- | --- |
|  | | Frequency | Percent | Valid Percent | Cumulative Percent |
| Valid |  | 539 | 96.6 | 96.6 | 96.6 |
|  | bean | 10 | 1.8 | 1.8 | 98.4 |
|  | cabbage | 1 | .2 | .2 | 98.6 |
|  | kale | 6 | 1.1 | 1.1 | 99.6 |
|  | lentile | 1 | .2 | .2 | 99.8 |
|  | potato | 1 | .2 | .2 | 100.0 |
|  | Total | 558 | 100.0 | 100.0 |  |

| **Ingredients of stew** | | | | | |
| --- | --- | --- | --- | --- | --- |
|  | | Frequency | Percent | Valid Percent | Cumulative Percent |
| Valid |  | 539 | 96.6 | 96.6 | 96.6 |
|  | oil,salt | 4 | .7 | .7 | 97.3 |
|  | oil,salt,onion | 2 | .4 | .4 | 97.7 |
|  | onion,oil,pepper,salt | 10 | 1.8 | 1.8 | 99.5 |
|  | onion,oil,pepper,salt,garlic | 1 | .2 | .2 | 99.6 |
|  | onion,oil,pepper,salt,tomato | 1 | .2 | .2 | 99.8 |
|  | onion,oil,salt,green pepper | 1 | .2 | .2 | 100.0 |
|  | Total | 558 | 100.0 | 100.0 |  |

| **type of stew** | | | | | |
| --- | --- | --- | --- | --- | --- |
|  | | Frequency | Percent | Valid Percent | Cumulative Percent |
| Valid |  | 558 | 100.0 | 100.0 | 100.0 |

| **Ingredients of stew** | | | | | |
| --- | --- | --- | --- | --- | --- |
|  | | Frequency | Percent | Valid Percent | Cumulative Percent |
| Valid |  | 557 | 99.8 | 99.8 | 99.8 |
|  | butter,oil,pepper,salt | 1 | .2 | .2 | 100.0 |
|  | Total | 558 | 100.0 | 100.0 |  |

| **type of stew** | | | | | |
| --- | --- | --- | --- | --- | --- |
|  | | Frequency | Percent | Valid Percent | Cumulative Percent |
| Valid |  | 523 | 93.7 | 93.7 | 93.7 |
|  | bean | 4 | .7 | .7 | 94.4 |
|  | bean, dark green lettuce | 1 | .2 | .2 | 94.6 |
|  | bean, kale | 1 | .2 | .2 | 94.8 |
|  | bean, potato | 1 | .2 | .2 | 95.0 |
|  | full,egg | 1 | .2 | .2 | 95.2 |
|  | kale | 9 | 1.6 | 1.6 | 96.8 |
|  | lentie,potato | 1 | .2 | .2 | 97.0 |
|  | lentil | 3 | .5 | .5 | 97.5 |
|  | lentil,bean | 4 | .7 | .7 | 98.2 |
|  | poatato | 1 | .2 | .2 | 98.4 |
|  | potato | 5 | .9 | .9 | 99.3 |
|  | potato, bean | 1 | .2 | .2 | 99.5 |
|  | potato, lentil | 2 | .4 | .4 | 99.8 |
|  | soas | 1 | .2 | .2 | 100.0 |
|  | Total | 558 | 100.0 | 100.0 |  |

| **Ingredients of stew** | | | | | |
| --- | --- | --- | --- | --- | --- |
|  | | Frequency | Percent | Valid Percent | Cumulative Percent |
| Valid |  | 522 | 93.5 | 93.5 | 93.5 |
|  | bean,oil,onion,garlic,green pepper,salt | 1 | .2 | .2 | 93.7 |
|  | oil, onion, salt, pepper | 1 | .2 | .2 | 93.9 |
|  | oil,salt | 10 | 1.8 | 1.8 | 95.7 |
|  | onion, oil, fenugreek, spice, salt, pepper | 1 | .2 | .2 | 95.9 |
|  | onion, oil, pepper, salt | 7 | 1.3 | 1.3 | 97.1 |
|  | onion, oil, salt | 1 | .2 | .2 | 97.3 |
|  | onion, oil, salt, pepper | 8 | 1.4 | 1.4 | 98.7 |
|  | onion, oil, salt, pepper, garlic | 1 | .2 | .2 | 98.9 |
|  | onion,oil,pepper,salt | 3 | .5 | .5 | 99.5 |
|  | onion,oil,pepper,salt,garlic,ginger | 1 | .2 | .2 | 99.6 |
|  | onion,oil,pepper,salt,garlic,ginger, | 1 | .2 | .2 | 99.8 |
|  | onion,oil,salt,pepper,garlic,ginger | 1 | .2 | .2 | 100.0 |
|  | Total | 558 | 100.0 | 100.0 |  |
