# Supplementary material for: Maternal dietary practices, dietary diversity, and nutrient composition of diets of lactating mothers in Jimma Zone, Southwest Ethiopia
Source: PLoS One. 2021 Jul 12;16(7):e0254259. doi: 10.1371/journal.pone.0254259 (PMC8274933; doi:10.1371/journal.pone.0254259)
Supplement: Attachment — Submitted filename: PONE-D-20-19141_reviewer.docx [file pone.0254259.s004.docx]

**PLOS ONE**

**Maternal dietary practices, dietary diversity, and nutrient composition of Diets of Lactating Mothers in Jimma Zone, Southwest Ethiopia**

--Manuscript Draft--

| **Manuscript Number:** | PONE-D-20-19141 |
| --- | --- |
| **Article Type:** | Research Article |
| **Full Title:** | Maternal dietary practices, dietary diversity, and nutrient composition of Diets of Lactating Mothers in Jimma Zone, Southwest Ethiopia |
| **Short Title:** | Mothers' diets in southwest Ethiopia |
| **Corresponding Author:** | Sirawdink Fikreyesus Forsido, Ph.D. Jimma University  JIMMA, OROMIA ETHIOPIA |
| **Keywords:** | Dietary diversity score; dietary adequacy; food composition; lactation |
| **Abstract:** | Background: Optimal nutrition during lactation is essential for the well-being of the mother and the infant. Studies have shown that access to nutrient-rich foods during lactation is critical as little stores of nutrients can have adverse effects. This study aimed to investigate the diversity, composition, and nutrient adequacy of diets of lactating mothers in Southwest Ethiopia.  Methods: A community-based cross-sectional survey was carried out in three districts of Jimma Zone, Southwest Ethiopia, from March to May 2014. A stratified multistage sampling technique was used to select 558 lactating mothers. Data were collected using a structured interviewer-administered questionnaire. Average minimum dietary diversity for women (MDD-W) was computed from two 24-h recalls. A cut off value of 3.4 was used to classify the dietary diversity into low and moderate or high. The proximate, mineral and anti-nutritional compositions of 12 commonly consumed foods were analysed using standard methods. Nutrient adequacy ratio (NAR) and Mean adequacy ratio (MAR) of these foods were estimated.  Results: The mean (±SD) DDS of the study participants was 3.37±0.85. The prevalence of “low DDS” was significantly (P<0.05) higher among informally educated, rural mothers, who reside in poor households and cereal producing district. The proximate composition (g/100g) of the sampled foods ranged between 24.8-65.6, 7.6- 19.8, 2.1-23.1, 2.0-27, 1.0-21.2, 0.9-45.8, for moisture, protein, crude fat, crude fibre, total ash and total carbohydrate content, respectively. The calorific value ranged between 124.5-299.6 Kcal/100g. The mineral contents (mg/100g) ranged between 9.5- 52.5, 2.2-4.2, 42.6-318.2, 150.7-379.9 for iron, zinc, calcium and phosphorus, respectively. The anti-nutritional factors contents (mg/100g) ranged between 11.1-  178.9 for phytate and 3.7-315.9 for tannin. All the commonly consumed maternal foods were not sufficient to meet the energy, fat and protein requirements, (NAR<1). However, all diets provided adequate iron and majority of the cereal-based foods provided adequate carbohydrate and minerals. The overall nutrient adequacy was below the cut-off point for all food types.  Conclusions: The diets of lactating mothers in Southwest Ethiopia lack diversity and nutrient adequacy. A community-based nutrition education on the importance of diet diversity and nutrient intake during lactation based on a multi-sectoral approach is needed. |
| **Order of Authors:** | Sirawdink Fikreyesus Forsido, MSc |
|  | Frehiwot Tadesse |
|  | Tefera Belachew |
|  | Oliver Hensel |
| **Additional Information:** |  |
| **Question** | **Response** |
| **Financial Disclosure** | This research was funded by the German Federal Ministry of Education and Research (BMBF) [(https://www.bmbf.de/en/index.html)](http://www.bmbf.de/en/index.html)) within the framework of the GlobE |

| Enter a financial disclosure statement that describes the sources of funding for the work included in this submission. Review the [submission guidelines](http://journals.plos.org/plosone/s/submission-guidelines#loc-financial-disclosure-statement) for detailed requirements. View published research articles from [*PLOS ONE*](http://journals.plos.org/plosone/) for specific examples.  This statement is required for submission and **will appear in the published article** if the submission is accepted. Please make sure it is accurate. | initiative through the Reduction of Post-Harvest Losses and Value Addition in East African Food Value Chains (RELOAD) project. The funder had no role in study design, data collection and analysis, decision to publish, or preparation of the manuscript. |
| --- | --- |
| **Unfunded studies**  Enter: *The author(s) received no specific funding for this work.*  **Funded studies**  Enter a statement with the following details:   - Initials of the authors who received each award - Grant numbers awarded to each author - The full name of each funder - URL of each funder website - Did the sponsors or funders play any role in the study design, data collection and analysis, decision to publish, or preparation of the manuscript? - **NO** - Include this sentence at the end of your statement: *The funders had no role in study design, data collection and analysis, decision to publish, or preparation of the manuscript.* - **YES** - Specify the role(s) played. |  |
| * typeset |  |
| **Competing Interests**  Use the instructions below to enter a competing interest statement for this submission. On behalf of all authors, disclose any [competing interests](http://journals.plos.org/plosone/s/competing-interests) that could be perceived to bias this work—acknowledging all financial support and any other relevant financial or non- financial competing interests.  This statement **will appear in the published article** if the submission is accepted. Please make sure it is accurate. View published research articles from [*PLOS ONE*](http://journals.plos.org/plosone/) for specific examples. | We have no conflicts of interest to declare. |

| **NO authors have competing interests**  Enter: *The authors have declared that no competing interests exist.*  **Authors with competing interests**  Enter competing interest details beginning with this statement:  *I have read the journal's policy and the authors of this manuscript have the following competing interests: [insert competing interests here]* |  |
| --- | --- |
| * typeset |  |
| **Ethics Statement**  Enter an ethics statement for this submission. This statement is required if the study involved:   - Human participants - Human specimens or tissue - Vertebrate animals or cephalopods - Vertebrate embryos or tissues - Field research   Write "N/A" if the submission does not require an ethics statement.  General guidance is provided below. Consult the [submission guidelines](http://journals.plos.org/plosone/s/submission-guidelines#loc-guidelines-for-specific-study-types) for detailed instructions. **Make sure that all information entered here is included in the Methods section of the manuscript.** | Ethical clearance was obtained from the Research and Ethical Review Board of Jimma University. Permission to undertake the study was obtained from every relevant authority in Jimma Zone. Each study participant was briefed about the research and offered the opportunity to ask questions. Then oral informed consent was obtained from each participant before participation in the study, and data were kept confidential. The Ethical Review Board decided to have verbal consent, as the study was not an intervention. Besides, the data collection and consent process were randomly checked by the Ethical Review Board to ensure the ethical undertaking of the research. |

| **Format for specific study types**  **Human Subject Research (involving human participants and/or tissue)**   - Give the name of the institutional review board or ethics committee that approved the study - Include the approval number and/or a statement indicating approval of this research - Indicate the form of consent obtained (written/oral) or the reason that consent was not obtained (e.g. the data were analyzed anonymously)   **Animal Research (involving vertebrate animals, embryos or tissues)**   - Provide the name of the Institutional Animal Care and Use Committee (IACUC) or other relevant ethics board that reviewed the study protocol, and indicate whether they approved this research or granted a formal waiver of ethical approval - Include an approval number if one was obtained - If the study involved *non-human primates*, add *additional details* about animal welfare and steps taken to ameliorate suffering - If anesthesia, euthanasia, or any kind of animal sacrifice is part of the study, include briefly which substances and/or methods were applied   **Field Research**  Include the following details if this study involves the collection of plant, animal, or other materials from a natural setting:   - Field permit number - Name of the institution or relevant body that granted permission |  |
| --- | --- |
|  |  |
| **Data Availability**  Authors are required to make all data underlying the findings described fully available, without restriction, and from the time of publication. PLOS allows rare exceptions to address legal and ethical concerns. See the [PLOS Data Policy](http://journals.plos.org/plosone/s/data-availability) and [FAQ](http://journals.plos.org/plosone/s/data-availability#loc-faqs-for-data-policy) for detailed information. | No - some restrictions will apply |

| A Data Availability Statement describing where the data can be found is required at submission. Your answers to this question constitute the Data Availability Statement and **will be published in the article**, if accepted. |  |
| --- | --- |
| **Important:** Stating ‘data available on request from the author’ is not sufficient. If your data are only available upon request, select ‘No’ for the first question and explain your exceptional situation in the text box. |  |
| Do the authors confirm that all data underlying the findings described in their manuscript are fully available without restriction? |  |
| **Describe where the data may be found in full sentences. If you are copying our sample text, replace any instances of XXX with the appropriate details.**   - If the data are **held or will be held in a public repository**, include URLs, accession numbers or DOIs. If this information will only be available after acceptance, indicate this by ticking the box below. For example: *All XXX files are available from the XXX database (accession number(s) XXX, XXX.*). - If the data are all contained **within the manuscript and/or Supporting Information files**, enter the following: *All relevant data are within the manuscript and its Supporting Information files.* - If neither of these applies but you are able to provide **details of access elsewhere**, with or without limitations, please do so. For example:   *Data cannot be shared publicly because of [XXX]. Data are available from the XXX Institutional Data Access / Ethics Committee (contact via XXX) for researchers who meet the criteria for access to confidential data.*  *The data underlying the results presented in the study are available from (include the name of the third party* | N/A |

| *and contact information or URL).*   - This text is appropriate if the data are owned by a third party and authors do not have permission to share the data.   * typeset |  |
| --- | --- |
| Additional data availability information: | Tick here if your circumstances are not covered by the questions above and you need the journal’s help to make your data available. |

Manuscript	[Click here to access/download;Manuscript;Maternal dietary practices 27.06.20.edited.docx](https://www.editorialmanager.com/pone/download.aspx?id=26897610&amp;guid=b9ef2160-6425-4998-a7bd-93b63e45010b&amp;scheme=1)

# 1 Maternal Dietary Practices, Dietary Diversity, and Nutrient Composition of Diets of

2 **Lactating Mothers in Jimma Zone, Southwest Ethiopia**
3 Sirawdink Fikreyesus Forsido a, b*, Frehiwot Tadesse b, Tefera Belachew c, Oliver Hensel a
4 a Department of Agricultural and Biosystems Engineering, Faculty of Organic Agricultural
5 Sciences, University of Kassel, Nordbahnhofstraße 1a, 37213, Witzenhausen, Germany
6 b Department of Post-Harvest Management, Jimma University, P.O. Box 307, Jimma, Ethiopia
7 c Department of Population and Family Health, College of Health Sciences, Jimma University,
8 P.O. Box 1104, Jimma, Ethiopia
9 *Corresponding author:
10 Department of Agricultural and Biosystems Engineering, Faculty of Organic Agricultural
11 Sciences, University of Kassel, Nordbahnhofstraße 1a, 37213, Witzenhausen, Germany

12	Phone: +251912785207; +49 5542 98 160; Fax: +251471110934; +49 5542 98 1520; E-mail:

13 [sirawdink@gmail.com](mailto:sirawdink@gmail.com)

# 14 Abstract

15 **Background:** Optimal nutrition during lactation is essential for the well-being of the mother and
16 the infant. Studies have shown that access to nutrient-rich foods during lactation is critical as
17 little stores of nutrients can have adverse effects. This study aimed to investigate the diversity,
18 composition, and nutrient adequacy of diets of lactating mothers in Southwest Ethiopia.
19 **Methods:** A community-based cross-sectional survey was carried out in three districts of Jimma
20 Zone, Southwest Ethiopia, from March to May 2014. A stratified multistage sampling technique
21 was used to select 558 lactating mothers. Data were collected using a structured interviewer-
22 administered questionnaire. Average minimum dietary diversity for women (MDD-W) was computed from two
23 24-h recalls. A cut off value of 3.4 was used to classify the dietary diversity into low and
24 moderate or high. The proximate, mineral and anti-nutritional compositions of 12 commonly
25 consumed foods were analysed using standard methods. Nutrient adequacy ratio (NAR) and
26 Mean adequacy ratio (MAR) of these foods were estimated.
27 **Results:** The mean (±SD) DDS of the study participants was 3.37±0.85. The prevalence of “low
28 DDS” was significantly (P<0.05) higher among informally educated, rural mothers, who reside
29 in poor households and cereal producing district. The proximate composition (g/100g) of the

30	sampled foods ranged between 24.8-65.6, 7.6-19.8, 2.1-23.1, 2.0-27, 1.0-21.2, 0.9-45.8, for

31 moisture, protein, crude fat, crude fibre, total ash and total carbohydrate content, respectively.
32 The calorific value ranged between 124.5-299.6 Kcal/100g. The mineral contents (mg/100g)

33	ranged between 9.5-52.5, 2.2-4.2, 42.6-318.2, 150.7-379.9 for iron, zinc, calcium and

34 phosphorus, respectively. The anti-nutritional factors contents (mg/100g) ranged between 11.1-
35 178.9 for phytate and 3.7-315.9 for tannin. All the commonly consumed maternal foods were not
36 sufficient to meet the energy, fat and protein requirements, (NAR<1). However, all diets
37 provided adequate iron and majority of the cereal-based foods provided adequate carbohydrate
38 and minerals. The overall nutrient adequacy was below the cut-off point for all food types.
39 **Conclusions:** The diets of lactating mothers in Southwest Ethiopia lack diversity and nutrient
40 adequacy. A community-based nutrition education on the importance of dietary diversity and
41 nutrient intake during lactation based on a multi-sectoral approach is needed.
42 **Keywords:** Dietary diversity score; dietary adequacy; food composition; lactation

# 43 Introduction

44 Maternal undernutrition is a significant cause of morbidity and mortality in low- and middle-
45 income countries throughout the world and is estimated to affect 22% of the mothers in Ethiopia.
46 Ethiopia is still among countries with a high burden of maternal malnutrition (Central Statistical
47 Agency (CSA) [Ethiopia] & ICF, 2016). Mothers from low-income settings are amongst the
48 most vulnerable to undernutrition because their body’s nutrient reserve is severely affected due
49 to frequent pregnancies and lactation (Haileslassie, Mulugeta, & Girma, 2013). Lactating women
50 who do not get enough energy and nutrients in their diets risk maternal depletion

51	(FAO/WHO/UNU, 2004, 2007; FAO/WHO, 2004).

52 According to the United Nations Children's Fund (UNICEF) conceptual framework of
53 undernutrition, inadequate	dietary intake	is	an	immediate	cause	of	maternal	and child
54 undernutrition (Black et al., 2008). Based on nutrition recommendations, energy needs are 25%
55 higher during lactation, and protein needs are 54% higher during lactation compared to a non-
56 lactating woman (Kominiarek & Rajan, 2016; Raymond, Kassim, Rose, & Agaba, 2018).
57 However, lactating women from low-income settings are considered as a nutritionally vulnerable
58 group because their dietary practices may not be optimal.
59 One of the available tools for measuring dietary adequacy among lactating mothers is dietary
60 diversity (Arimond, Torheim, Wiesmann, Joseph, & Carriquiry, 2008). However, literature
61 evidenced that dietary diversity among lactating mothers is very low in low-income settings
62 (Nguyen et al., 2013). Several demographic, socio-economic, and other factors, which could vary
63 from setting to setting, could contribute to reduced dietary diversity (Weldehaweria et al., 2016).
64 Another gap that is noticed regarding maternal diets in low-income settings like Ethiopia is the
65 composition of the foods customarily consumed is not exhaustively documented in the food
66 composition tables. This information is fundamental for programmes which operate in the
67 agriculture-nutrition-health interface. Inadequate food composition data may lead to a failure in
68 understanding the relationship between nutrient intake and health or result in inappropriate,
69 inefficient interventions to improve micronutrient status (Hulshof et al., 2019).
70 Information on dietary practices, dietary diversity and associated factors, and composition of
71 foods of lactating mothers are urgently needed for prioritizing, designing and initiating
72 intervention programs aiming at improving maternal and child nutrition. However, little is
73 documented on maternal dietary practices, dietary diversity and the associated factors and
74 composition of maternal foods in the study area. The objective of this study was therefore to
75 assess the dietary practices, diversity, composition, and nutrient adequacy of diets of lactating
76 mothers in Jimma zone, Southwest Ethiopia.

# 77 Methods

78 **Setting and study design**
79 The study was conducted in Southwest Ethiopia, Jimma zone. Three districts were purposively
80 selected based on their agricultural production. Omo Nada, Dedo and Mana are cereal, vegetable
81 and cash crop producing areas, respectively. The survey was a community-based cross-sectional
82 study.

# 83 Participants, sample size and sampling technique

84 The study population included all lactating mothers in the study area. From these, 558 mothers
85 were sampled (Faul, Erdfelder, Lang, & Buchner, 2007) following a stratified multistage
86 sampling procedure. On the first stage, the districts were stratified into lowland, midland and
87 highland agroecology. Out of the 19 districts in the zone, three (1 Lowland, 1 Midland and 1
88 Highland) were selected purposively. Then, out of all the kebeles in the three districts, six rural
89 and three urban kebeles were sampled by lottery method. In these selected kebeles, households
90 with lactating mothers were systematically identified using the registration at the health posts
91 (February 2014). The calculated sample (558) was non-proportionally allocated to the selected
92 districts (186 for each) then to the selected kebeles (62 for each kebele). A simple random
93 sampling of the households was employed to recruit the study subjects. Sick mothers were
94 excluded from the sample. Participants were recruited March to May 2014.

# 95 Variables

96 The variables involved in the analysis include socio-economic and demographic factors. These
97 factors included family composition, age, household size, highest educational level attained by
98 mothers and fathers, the primary occupation of mothers and fathers, the wealth of the household
99 and source of income, source of drinking water and availability of toilet facility, availability of
100 health centres and education or training given on health and nutrition. Additionally, maternal
101 dietary practices, maternal dietary diversity, nutrient compositions of maternal diets and nutrient
102 adequacy of maternal diets were also involved in the analysis.

# 103 Measurements

104 **Maternal dietary practices**
105 The guideline on essential nutrition actions for healthy maternal nutrition during breastfeeding
106 was followed to assess dietary practices of the mothers. Knowledge and practice in control of
107 maternal iron deficiency anaemia, adequate food-intake (namely increased energy intake through
108 two additional meals a day, improved variety, daily use of iodized salt for all family members)
109 and birth spacing were assessed (World Health Organization, 2013). Semi-structured
110 questionnaires were used to collect data on demographic, socio-economic and dietary practices.

# 111 Dietary diversity

112 A single 24-h dietary recall was used to obtain data on dietary diversity. Dietary diversity was
113 assessed with a scale of nine food groups namely starchy staples, dark green leafy vegetables,
114 another vitamin A rich fruits and vegetables, other fruits and vegetables, fats and oils, meat and
115 fish, eggs, legumes, nuts and seeds and milk and milk products (Arimond et al., 2010). Dietary
116 diversity score (DDS) was found to be moderate or high when a mother consumed greater than
117 3.4 mean food groups per day and respondents with <3.4 mean food groups consumed were
118 considered as having lower dietary diversity (Weldehaweria et al., 2016).

# 119 Nutrient composition of maternal diets

120 Based on the responses on maternal dietary practices, twelve customary foods consumed by the
121 mothers were selected for composition analysis. Composite samples were collected, dried,
122 ground and packaged (Pomeranz & Meloan, 2000). These samples were analysed for proximate
123 composition (protein, fat, carbohydrate, moisture, ash and fibre), energy content, mineral (iron,
124 zinc, calcium and phosphorous) and anti-nutritional factors (phytate and tannin) following the
125 respective standard methods of analysis (AOAC International, 2005).

# 126 Nutrient adequacy of maternal diets

127 According to Public Health Department of Georgia and World Health Organization (2005), the
128 average recommended gram of portion per day is 550g/day, 450g/day and 175g/day for cereal,
129 vegetable and legume food types, respectively for a healthy life. Nutrient intake was calculated
130 as a multiple of recommended gram of portion per day and composition (proximate and mineral)
131 of the food as indicated in the equation below.

𝑔	𝑔	𝑔

132	𝑁𝑢𝑡𝑟𝑖𝑒𝑛𝑡 𝑖𝑛𝑡𝑎𝑘𝑒 (𝑑𝑎𝑦) = 𝑅𝑒𝑐𝑜𝑚𝑚𝑒𝑛𝑑𝑒𝑑 𝑝𝑜𝑟𝑡𝑖𝑜𝑛 (𝑑𝑎𝑦) ∗ 𝑁𝑢𝑡𝑟𝑖𝑒𝑛𝑡 𝑐𝑜𝑚𝑝𝑜𝑠𝑖𝑡𝑖𝑜𝑛 (100𝑔)

133 Nutrient adequacy ratio (NAR) was calculated as the ratio of a subject's nutrient intake to the
134 estimated average requirement calculated using the Food and Agriculture Organization/ World
135 Health Organization recommended nutrient intakes for mothers (FAO/WHO/UNU, 2004, 2007;
136 FAO/WHO, 2004). The mean adequacy ratio (MAR) was calculated as the sum of NARs for all
137 evaluated nutrients divided by the number of assessed nutrients, expressed as a percentage
138 (Steyn, Nel, Nantel, Kennedy, & Labadarios, 2006) as indicated in the equations below.

𝑁𝐴𝑅 =

𝐴𝑐𝑡𝑢𝑎𝑙 𝑖𝑛𝑡𝑎𝑘𝑒 𝑜𝑓 𝑎 𝑛𝑢𝑡𝑟𝑖𝑒𝑛𝑡 𝑝𝑒𝑟 𝑑𝑎𝑦

𝑅𝑒𝑐𝑜𝑚𝑚𝑒𝑛𝑑𝑒𝑑 𝐷𝑎𝑖𝑙𝑦 𝐴𝑙𝑙𝑜𝑤𝑎𝑛𝑐𝑒 𝑜𝑓 𝑡ℎ𝑒 𝑛𝑢𝑡𝑟𝑖𝑒𝑛𝑡

𝑀𝐴𝑅 =

∑ 𝑁𝐴𝑅 (𝑒𝑎𝑐ℎ 𝑡𝑟𝑢𝑛𝑐𝑎𝑡𝑒𝑑 𝑎𝑡 1)

𝑁𝑢𝑚𝑏𝑒𝑟 𝑜𝑓 𝑛𝑢𝑡𝑟𝑖𝑒𝑛𝑡𝑠

# 141 Statistical analysis

142 Data were entered, cleaned and then analyzed using SPSS version 20. Descriptive summaries
143 using frequencies and proportions were used to present the study results. In this study, the
144 dependent variable was dietary diversity coded one as lower dietary diversity and 0 as moderate
145 or high dietary diversity. Descriptive statistics such as mean, median, range and percentages
146 were calculated. Chi-square test was used to see the association between dietary diversity and
147 socio-economic and demographic variables. For all statistical analyses, a P value < 0.05 was
148 considered for statistical significance.

# 149 Ethics approval and consent to participate

150 Ethical clearance was obtained from the Research and Ethical Review Board of Jimma
151 University. Permission to undertake the study was obtained from every relevant authority in
152 Jimma Zone. Each study participant was briefed about the research and offered the opportunity
153 to ask questions. Then oral informed consent was obtained from each participant before
154 participation in the study, and data were kept confidential. The Ethical Review Board decided to
155 have verbal consent, as the study was not intervention. Besides, the data collection and consent
156 process were randomly checked by the Ethical Review Board to ensure the ethical undertaking of
157 the research.

# 159 Results

160 **Characteristics of the sample**
161 A total of 558 mothers who had children aged 0-24 months were interviewed, and all agreed to
162 participate in the study, which made the response rate of 100%. Majority 372 (66.7) of the
163 participants were from the rural part of the survey area, Muslims in religion 516 (92.5%), Oromo
164 in ethnicity 486 (87.1%), married and living together 501(89.8%) and 336 (60.2%) were
165 uneducated. A large proportion of the families belong to lower middle class or socio-economic
166 status because most of them were engaged in small business, and many 436 (78.1%) were
167 housewives (Table 1).

# 168 Maternal dietary practices

169 Majority of the lactating mothers reported that they received the following health information:
170 have two additional meals a day (84.1%), consume a variety of food groups (82.8%), use
171 insecticide-treated net (94.4%), and family planning (97.5%). On the contrary, only 39.1% of the
172 participants reported receiving information to take iodized salt daily. When it comes to the
173 practice, only 22.2% of the respondents consumed two additional meals per day, 14.5% reported
174 consumption of a variety of food groups during lactation, and 36% reported the use of iodized salt
175 in daily basis. Two-thirds of the mothers (65.6%) used bed net to protect themselves from malaria, and
176 61.5% of the mothers reported practising family planning (Table 2).

# 177 Dietary diversity

178 Table 3 summarises the dietary diversity score and percentage of women consuming each of the
179 nine food groups. Among the nine food groups, the mean ± SD DDS for the study participants
180 was 3.37±0.85 with a maximum score of 6 and a minimum score of 0. More than half of the
181 participants (54.5%) had low DDS whereas 45.5% had moderate or high DDS. Results of the 24-
182 hour recall showed that almost all of the mothers consume starchy staples (99.1%) and nearly
183 three-fourth (77.2%) of the mothers consumed legumes and nuts. Only 13.4% of the participants
184 consumed dairy products, and none of them consumed
185 organ meat while only 3.2% consumed other meats or flesh foods and 6.1% consumed eggs. Half
186 (50.4%) of the respondents reported consumption of vitamin A-rich leafy green vegetables. The
187 use of vitamin A-rich fruits and vegetables was low (3.4%). However, most of the respondents
188 (89.4%) reported consumption of other fruits and vegetables.
189 Table 4 shows the association between socio-demographic variables and maternal dietary
190 diversity. This study showed the distribution of dietary diversity over different influencing
191 factors. Accordingly, dietary diversity score was significantly (P<0.01) affected by districts,
192 residence area, age, educational status and the socio-economic situation of the household.
193 Mothers who live in cereal producing community and rural villages, who are in 25-29 years age
194 group, illiterate and poor mothers had lower DDS as compared with their respective
195 counterparts. Rural mothers exhibited significantly (p<0.01) more substantial proportion (59.1%)
196 of low DDS compared to their urban (42.5%) counterparts. The prevalence of low DDS was high
197 in the cereal producing district (64.2%) than in vegetable producing (50.0%) or cash crop
198 producing (46.2%) districts.

# 199 Nutrient composition of maternal diets

200 The proximate composition and calorific value of the sampled maternal foods ranged between

201	24.8–65.6g/100g, 7.6–19.8 g/100g, 2.1–23.1 g/100g, 2.0–27 g/100g, 1.0–21.2 g/100g, 0.9–45.8

202	g/100g and 124.5–299.6Kcal/100g for moisture, protein, crude fat, crude fibre, total ash, total

203	carbohydrate and energy content, respectively (Table 5). The mineral contents ranged between

204	9.5–52.5mg/100g, 2.2–4.2mg/100g, 42.6–318.2mg/100g, 150.7–379.9mg/100g for iron, zinc,

205 calcium, and phosphorus, respectively. The anti-nutritional factors contents ranged between
206 11.1–178.9mg/100g for phytate and 3.7–315.9mg/100g for tannin (Table 6).

# 207 Nutrient adequacy of maternal diets for achieving maternal nutritional goals

208 Out of the twelve foods assessed in this study, most of the maternal diets did not contain
209 adequate protein, fat and energy (NAR<1). Most of the cereal-based foods contain sufficient
210 minerals (NAR>1), and fermentation is believed to have played its role for this. The overall
211 adequacy is less than 1 for all diets (MAR<1) (Table 7).

# 212 Discussion

213 **Maternal dietary practices**
214 Majority of the study participants did not change their previous food intake habit during lactation
215 and eat the same quantity and quality (diversity) of the meal as they were not lactating, which is
216 against the essential nutrition actions to improve maternal health and nutrition (World Health
217 Organization, 2013). It is known that a lactating mother should produce about 700 to 800ml of
218 milk per day and this requires an additional energy need of about 550 calories (two extra meals)
219 per day (FAO/WHO/UNU, 2004; Haileslassie et al., 2013; Ogechi, 2014). Our finding on the
220 practice of consumption of iodized salt daily was lower than 76.3% reported in the capital city,
221 Addis Ababa (Bazezew, Yallew, & Belew, 2018). This might be due to the accessibility of
222 different media nearby for the target group in the study area.

# 223 Dietary Diversity

224 The women's DDS was similar to those previously reported in Bangladeshi women as well as in
225 women in Burkina Faso, Mali, Mozambique, Bangladesh, and the Philippines using the same
226 definition of dietary diversity (Arimond et al., 2010). Mothers who lived in the grain-producing
227 district and rural villages, who are in 25-29 years age group, illiterate and poor mothers had
228 lower DDS as compared with their respective counterparts. These results are in agreement with
229 previous reports which indicated that there is a positive relationship between dietary diversity
230 and diversified agricultural production and income from the agricultural product (Gonder, 2011),
231 being a younger mother (Ogechi, 2014), and formal education to the mother (Ajani, 2010). In the
232 cash crop producing district, mothers could have enough money for household expenditure to
233 buy various food commodities. They might have a diverse diet compared to mothers living in
234 cereal producing regions. Therefore, the study participants highly rely on a few staple foods and
235 often include little or no animal products and few fresh fruits and vegetables as compared to
236 Dedo and Mana districts. This finding is consistent with previous reports (Ajani, 2010; Gonder,
237 2011; Ruel, 2003) who stated that diversified agricultural production and income from
238 agricultural product market even in a more impoverished household has a positive relationship
239 with dietary diversity.
240 Similarly, DDS was significantly different among lactating mothers placed in the separate
241 residence. A large proportion (59.1%) of mothers in the rural area showed a low DDS than an
242 urban residential area. Previous findings by (Ruel, 2003) also indicated urban households had a
243 consistently higher dietary diversity than rural households. Although in contrast, higher DDS
244 was reported in the rural sector (Ajani, 2010).
245 According to (Ajani, 2010) educational status and household wealth were interrelated had a
246 positive association with dietary diversity, because higher education attainment is likely to be
247 associated with a higher income so as the mothers would have money to buy different food
248 commodity for household food preparation.

# 249 Composition of maternal diets

250 Considering the average recommended gram of portion per day, 550g/day, 450g/day and
251 175g/day for cereal, vegetable and legume food types, respectively, only kale sauce can fulfil the
252 RDA for protein (17.33g/day) (FAO/WHO/UNU, 1981, 2004). Whereas only unleavened bread
253 made of a blend of wheat and corn and *Injera* made of teff can fulfil the RDA for carbohydrate
254 (250 g/day) (FAO/WHO/UNU, 2004). Concerning the fibre content, four out of twelve food
255 types fulfil the RDA. These are kale sauce, *Injera* made of teff, maize and sorghum, *Injera* made
256 of teff, maize and rice and, *Injera* made of teff and maize (FAO/WHO/UNU, 1981, 2004). Even
257 though there is no recommendation of ash for lactating mothers, it is known that as ash content is
258 correlated with micronutrient content in that particular food type. Therefore, from the 12 food
259 types analysed, kale sauce could provide relatively healthy micronutrients to the lactating
260 mothers. Based on the recommended gram of portion for adults none of the 12 food types fulfils
261 the RDA for fat (69.1 g/day) (FAO/WHO/UNU, 2004), and energy (2750 kcal/day)

262	(FAO/WHO/UNU, 1981).

263 Among the mineral forms analysed, iron was predominantly found in all tested foods above the
264 recommended amount (10mg/day). Except for lentil, bean and pea powder sauces, all the food
265 types can fulfil the recommended daily allowance for zinc (12mg/day). All *Injera* types and the
266 unleavened bread made of maize can fulfil the RDA for Ca. Except for lentil, bean and pea
267 powder sauces and *Injera* made of blends of teff, sorghum, and rice, all the food types can fulfil
268 the recommended daily allowance for P (1000mg/day) (FAO/WHO/UNU, 2004).
269 High phytate intakes and low fruit consumption may compromise iron, zinc, and to a lesser
270 extent calcium status in these mothers (Lopez, Leenhardt, Coudray, & Remesy, 2002). Tannins
271 had been reported to affect protein digestibility, adversely influencing the bioavailability of non-
272 haem iron leading to poor iron and calcium absorption (Delimont, Haub, & Lindshield, 2017).
273 There is no recommendation or cut-off points for the availability of anti-nutritional factors in
274 foods while reducing the anti-nutrition levels to the best minimum using different strategies is
275 advised (Samtiya, Aluko, & Dhewa, 2020).

# 276 Nutrient adequacy of maternal diets for achieving maternal nutritional goals

277 According to (Ongosi, 2011), the ideal cut-off for nutrient adequacy should be 1, which would
278 mean that all the nutrients were consumed in a sufficient amount. In another way, the NAR for
279 protein and calorific value/energy throughout the whole sampled food types were found to be
280 below 1, which means that all the commonly eaten foods by lactating mothers were not sufficient
281 to meet the protein and energy requirements. Protein from cereals is usually with relatively low
282 digestibility and quality (Swaminathan, Vaz, & Kurpad, 2012). The consequences of low energy
283 in the diets is the women suffer from chronic energy deficiency and will have poor nutritional
284 status (Haileslassie et al., 2013). Lack of dietary diversification and the foods lack vegetables,
285 fruits, and animal source food types (Ruel, 2003). The overall nutrient adequacy was different
286 for the twelve types of food types. MAR was below the cut-off point for all food types.
287 As presented in Table 7, most of the food types had sufficient mineral content which had NAR
288 above 1. These could be, from the supply side, because of Ethiopian foods, especially *Injera*, had
289 a significantly high amount of minerals (Haileslassie et al., 2013). The other reason, from a
290 demand side, could be since breastfeeding usually suppresses menstruation for a few months,
291 lactating mothers iron requirement is decreased to 10 mg/day RDA (Ongosi, 2011). Care should
292 be taken in interpreting these results as bioaccessibility and bioavailability of minerals from
293 plant-based foods is impaired (Rousseau, Kyomugasho, Celus, Hendrickx, & Grauwet, 2020).

# 294 Limitations and strengths of the study

295 **Limitation of the study**
296 Recall bias. Over and under-reporting of maternal dietary intakes. Dietary intakes of the mothers
297 were not directly measured by using portion sizes as family members in Ethiopia consume food
298 from a common plate. Ethiopia does not have a food guide; therefore we used the amount of
299 recommended portions per day (in grams) for Georgia. Vitamin contents of the meals were not
300 analysed.

# 301 Strength of the study

302 Maternal diets, whose composition can not be obtained from the Food Composition Tables, were
303 analysed.

# 304 Conclusions

305 Our study shows that lactating women in Southwest Ethiopia consume diets that lack sufficient
306 nutrient-dense foods to achieve micronutrient adequacy. The dietary diversity of lactating
307 mothers in the study area was not satisfactory. Kale sauce, which is traditionally popular in
308 South and Southwest part of rural Ethiopia, owes a nutritional favour to these community. A
309 community-based nutritional education on food diversification and local processing techniques
310 based on a multi-sectoral approach is needed to curb the problem of malnutrition among lactating
311 mothers in the study area.

# 312 Acknowledgements

313 We are grateful to the mothers for their participation and the health extension workers for their
314 assistance. The authors also thank Ethiopian Public Health Institute for assisting in the nutrient
315 analysis.

# 316 Declarations

317 **Consent for publication**
318 Not applicable

# 319 Availability of data and materials

320 The datasets supporting the conclusions of this article are included in the article.

# 321 Competing interests

322 We have no conflicts of interest to declare.

# 323 Funding

324 This research was funded by the German Federal Ministry of Education and Research (BMBF)
325 (<https://www.bmbf.de/en/index.html>) within the framework of the GlobE initiative through the
326 Reduction of Post-Harvest Losses and Value Addition in East African Food Value Chains
327 (RELOAD) project. The funders had no role in study design, data collection and analysis,
328 decision to publish, or preparation of the manuscript.

# 329 Authors' contributions

330 Conceived and designed the experiments: SFF TB OH. Collected the data: FT SFF. Analyzed the
331 data: SFF FT TB. Contributed reagents/materials/analysis tools: SFF TB OH. Prepared draft
332 manuscript: SFF. Finalized the manuscript and provided a critical review: TB OH. All authors
333 have read and approved the final manuscript.

# 334 References

335 Ajani, S. R. (2010). An Assessment of Dietary Diversity in Six Nigerian States. *Afr. J. Biomed.*

336	*Res*, *13*, 161–167.

337 AOAC International. (2005). *Official methods of analysis of AOAC International* (18th ed.).
338 Gaithersburg, MD, USA: AOAC International.
339 Arimond, M., Torheim, L. E., Wiesmann, M., Joseph, M., & Carriquiry, A. (2008). Dietary
340 diversity as a measure of women’s diet quality in resource-poor areas: Results from rural
341 Bangladesh site. *Washington, DC: Food and Nutrition Technical Assistance (FANTA) and*
342 *Project/Academy for Educational Development (AED)*.
343 Arimond, M., Wiesmann, D., Becquey, E., Carriquiry, A., Daniels, M. C., Deitchler, M., …

| 344 | Torheim, L. E. (2010). Simple Food Group Diversity Indicators Predict Micronutrient |
| --- | --- |
| 345 | Adequacy of Women’s Diets in 5 Diverse, Resource-Poor Settings. *The Journal of* |
| 346 | *Nutrition*, *140*(11), 2059S-2069S. https://doi.org/10.3945/jn.110.123414 |
| 347 | Bazezew, M. M., Yallew, W. W., & Belew, A. kassahun. (2018). Knowledge and practice of |
| 348 | iodized salt utilization among reproductive women in Addis Ababa City. *BMC Research* |

| 349 | *Notes*, *11*(1), 734. https://doi.org/10.1186/s13104-018-3847-y |
| --- | --- |
| 350 | Black, R. E., Allen, L. H., Bhutta, Z. a., Caulfield, L. E., de Onis, M., Ezzati, M., … Rivera, J. |
| 351 | (2008). Maternal and child undernutrition: global and regional exposures and health |
| 352 | consequences. *The Lancet*, *371*(9608), 243–260. https://doi.org/10.1016/S0140- |
| 353 | 6736(07)61690-0 |
| 354 | Central Statistical Agency (CSA) [Ethiopia], & ICF. (2016). *Ethiopia Demographic and Health* |
| 355 | *Survey 2016*. Addis Ababa, Ethiopia, and Rockville, Maryland, USA. Retrieved from |
| 356 | https://dhsprogram.com/pubs/pdf/FR328/FR328.pdf |
| 357 | Delimont, N. M., Haub, M. D., & Lindshield, B. L. (2017). The Impact of Tannin Consumption |
| 358 | on Iron Bioavailability and Status: A Narrative Review. *Current Developments in Nutrition*, |
| 359 | *1*(2), 1–12. https://doi.org/10.3945/cdn.116.000042 |
| 360 | FAO/WHO/UNU. (1981). *Diet and the Pregnant and Lactating Woman. A report of a Joint* |
| 361 | *FAO/WHO/UNU Expert Consultation on Energy and Protein Requirements.* Rome. |
| 362 | Retrieved from [www.fao.org/3/a-m2998e/](http://www.fao.org/3/a-m2998e/) |
| 363 | FAO/WHO/UNU. (2004). *Human energy requirements* (FOOD AND NUTRITION |
| 364 | TECHNICAL REPORT SERIES No. 1). Rome, Italy: World Health Organization. |
| 365 | Retrieved from |
| 366 | <http://www.who.int/nutrition/publications/nutrientrequirements/9251052123/en/> |
| 367 | FAO/WHO/UNU. (2007). *Protein and amino acid requirements in human nutrition* (Technical |
| 368 | Report Series No. 935). |
| 369 | FAO/WHO. (2004). *Vitamin and mineral requirements in human nutrition*. (World Health |

| 370 | Organization, Ed.) (2nd editio). Rome: WHO. |
| --- | --- |
| 371 | Faul, F., Erdfelder, E., Lang, A.-G., & Buchner, A. (2007). G*Power 3: a flexible statistical |
| 372 | power analysis program for the social, behavioral, and biomedical sciences. *Behavior* |
| 373 | *Research Methods*, *39*, 175–191. https://doi.org/10.3758/BF03193146 |
| 374 | Gonder, C. E. (2011). *Is subsistence enough? Examining the impact of household farm* |
| 375 | *biodiversity in Bukidnon, Philippines*. University of Illinois at Urbana-Champaign. |
| 376 | Haileslassie, K., Mulugeta, A., & Girma, M. (2013). Feeding practices, nutritional status and |
| 377 | associated factors of lactating women in Samre Woreda, South Eastern Zone of Tigray, |
| 378 | Ethiopia. *Nutrition Journal*, *12*(28). https://doi.org/10.1186/1475-2891-12-28 |
| 379 | Hulshof, P., Doets, E., Seyha, S., Bunthang, T., Vonglokham, M., Kounnavong, S., … Brouwer, |
| 380 | I. (2019). Food Composition Tables in Southeast Asia: The Contribution of the SMILING |
| 381 | Project. *Maternal and Child Health Journal*, *23*(1), 46–54. https://doi.org/10.1007/s10995- |
| 382 | 018-2528-8 |
| 383 | Kominiarek, M. A., & Rajan, P. (2016). Nutrition Recommendations in Pregnancy and |
| 384 | Lactation. *The Medical Clinics of North America*, *100*(6), 1199–1215. |
| 385 | https://doi.org/10.1016/j.mcna.2016.06.004 |
| 386 | Lopez, H. W., Leenhardt, F., Coudray, C., & Remesy, C. (2002). Minerals and phytic acid |
| 387 | interactions: is it a real problem for human nutrition? *International Journal of Food Science* |
| 388 | *and Technology*, *37*(7), 727–739. https://doi.org/10.1046/j.1365-2621.2002.00618.x |
| 389 | Nguyen, P. H., Avula, R., Ruel, M. T., Saha, K. K., Ali, D., Tran, L. M., … Rawat, R. (2013). |
| 390 | Maternal and child dietary diversity are associated in Bangladesh, Vietnam, and Ethiopia. |

391	*The Journal of Nutrition*, *143*(7), 1176–1183. https://doi.org/10.3945/jn.112.172247

392	Ogechi, U. P. (2014). A Study of the Nutritional Status and Dietary Intake of Lactating Women

393	in Umuahia, Nigeria. *American Journal of Health Research*, *2*(1), 20.

394	https://doi.org/10.11648/j.ajhr.20140201.14

395 Ongosi, A. N. (2011). *Nutrient intake and nutrition knowledge of lactating women (0-6) months*
396 *postpartum) in a low socio-economic area in Nairobi, Kenya*. University of Pretoria.
397 Retrieved from https://repository.up.ac.za/handle/2263/25935
398 Pomeranz, Y., & Meloan, C. E. (2000). *Food Analysis: Theory and Practice* (Third).
399 Gaithersburg, Maryland: ASpern Publishers, Inc.
400 Public Health Department of Georgia, & World Health Organization. (2005). *Healthy eating –*
401 *the main key to health*. Retrieved f[rom http://www.fao.org/3/a-as682e.pdf](http://www.fao.org/3/a-as682e.pdf)
402 Raymond, J., Kassim, N., Rose, J. W., & Agaba, M. (2018). Optimal dietary patterns designed
403 from local foods to achieve maternal nutritional goals. *BMC Public Health*, *18*(1), 451.

404	https://doi.org/10.1186/s12889-018-5369-x

405	Rousseau, S., Kyomugasho, C., Celus, M., Hendrickx, M. E. G., & Grauwet, T. (2020, March 8).

406	Barriers impairing mineral bioaccessibility and bioavailability in plant-based foods and the

407	perspectives for food processing. *Critical Reviews in Food Science and Nutrition*. Taylor

408	and Francis Inc. https://doi.org/10.1080/10408398.2018.1552243

409	Ruel, M. T. (2003). Is Dietary Diversity an Indicator of Food Security or Dietary Quality? A

410	Review of Measurement Issues and Research Needs. *Food and Nutrition Bulletin*, *24*(2),

411	231–232. https://doi.org/10.1177/156482650302400210

412	Samtiya, M., Aluko, R. E., & Dhewa, T. (2020). Plant food anti-nutritional factors and their

413	reduction strategies: an overview. *Food Production, Processing and Nutrition*, *2*(1), 6.

414	https://doi.org/10.1186/s43014-020-0020-5

415	Steyn, N. P., Nel, J. H., Nantel, G., Kennedy, G., & Labadarios, D. (2006). Food variety and

416	dietary diversity scores in children: are they good indicators of dietary adequacy? *Public*

417	*Health Nutr*, *9*(5), 644–650. Retrieved from

418	<http://www.ncbi.nlm.nih.gov/pubmed/16923296>

419	Swaminathan, S., Vaz, M., & Kurpad, A. V. (2012). Protein intakes in India. *British Journal of*

420	*Nutrition*, *108*(S2), S50–S58. https://doi.org/DOI: 10.1017/S0007114512002413

421	Weldehaweria, N. B., Misgina, K. H., Weldu, M. G., Gebregiorgis, Y. S., Gebrezgi, B. H.,

422	Zewdie, S. W., … Alemu, W. (2016). Dietary diversity and related factors among lactating

423	women visiting public health facilities in Aksum town, Tigray, Northern Ethiopia. *BMC*

424	*Nutrition*, *2*(1), 38. https://doi.org/10.1186/s40795-016-0077-3

425	World Health Organization. (2013). Essential nutrition actions: improving maternal, newborn,

426	infant and young child health and nutrition.

427

428	Table 1 Maternal and household characteristics (Jimma Zone, Southwest Ethiopia 2014)

| **Characteristics** | **N** | **%** |
| --- | --- | --- |
| Maternal Age (Years) |  |  |
| 15-19 | 26 | 4.7 |
| 20-29 | 360 | 64.5 |
| 30-39 | 161 | 28.9 |
| 40-49 | 11 | 2 |
| Maternal education |  |  |
| Informal education | 338 | 60.6 |
| Formal education | 220 | 39.4 |
| Maternal Occupation |  |  |
| Housewife | 436 | 78.1 |
| Merchant | 83 | 14.9 |
| Other^§^ | 39 | 7 |
| Maternal Marital status |  |  |
| Single | 33 | 5.9 |
| Married | 501 | 89.8 |
| Separated | 16 | 2.9 |
| Widowed | 5 | 0.9 |
| Divorced | 3 | 0.5 |
| Religion |  |  |
| Muslim | 516 | 92.5 |
| Orthodox | 33 | 5.9 |
| Protestant | 9 | 1.6 |
| Residence |  |  |
| Rural | 372 | 66.7 |
| Urban | 186 | 33.3 |
| Number of children per mother |  |  |
| 1-2 children | 252 | 45.2 |
| 3 and above children | 306 | 54.8 |
| District |  |  |
| Cash crop-producing | 186 | 33.3 |
| Cereal producing | 186 | 33.3 |
| Vegetable producing | 186 | 33.3 |
| Family Size |  |  |
| 2-4 Members | 201 | 36 |
| 5-7 Members | 254 | 45.5 |
| 8-9 Members | 81 | 14.5 |
| 10 and Above Members | 22 | 3.9 |

Socio-economic Status

| Low | 186 | 33.3 |
| --- | --- | --- |
| Medium | 189 | 33.9 |
| High | 183 | 32.8 |
| Husband’s education |  |  |
| Informal education | 214 | 41.4 |
| Formal education | 303 | 58.6 |
| Husband’s Occupation |  |  |
| Farmer | 318 | 63.5 |
| Daily Laborer | 63 | 12.6 |
| Merchant | 59 | 11.8 |
| Other$ | 61 | 12.2 |
| Primary source of household income |  |  |
| Farming | 347 | 62.2 |
| Wage/Salary | 111 | 19.9 |
| Business | 90 | 16.1 |
| Salary | 44 | 7.9 |
| Other¥ | 10 | 1.8 |

^§^Farmer, government employee, NGO employee, daily labourer;

$Government Employee, NGO Employee, daily labourer

¥Pension, remittance, commission

429

430	Table 2 Nutrition during breastfeeding of lactating mothers in three districts of Jimma Zone,

431	Southwest Ethiopia

|  |  | **n** | **%** | **n** | **%** |
| --- | --- | --- | --- | --- | --- |
|  | Use insecticide-treated net | 527 | 94.4 | 366 | 65.6 |
|  | Consume two additional meals a day | 469 | 84.1 | 124 | 22.2 |
|  | Consume a variety of food groups | 462 | 82.8 | 81 | 14.5 |
|  | Daily use of iodized salt | 218 | 39.1 | 201 | 36.0 |
|  | Family Planning | 544 | 97.5 | 343 | 61.5 |
| 432 |  |  |  |  |  |

# Variables Educated Practised

433	Table 3 Dietary diversity score and percentage of women consuming each of the 9 food groups

|  | |  | DDS |  |
| --- | --- | --- | --- | --- |
|  |  | Mean | 3.37 |  |
|  |  | Median | 3 |  |
|  |  | SD | 0.85 |  |
|  |  | Minimum | 0 |  |
|  |  | Maximum | 6 |  |
|  |  | Women dietary diversity  Low | N (%)  304 (54.5) |  |
|  |  | Moderate or high  Consumption | 254 (45.5)  N (%) |  |
|  |  | Starchy staples | 553 (99.1) |  |
|  |  | Legumes and nuts | 431 (77.2) |  |
|  |  | Dairy products | 75 (13.4) |  |
|  |  | Organ meats | 0 (0) |  |
|  |  | Other meats/flesh foods | 18 (3.2) |  |
|  |  | Eggs | 34 (6.1) |  |
|  |  | Vitamin A-rich leafy green vegetables | 281 (50.4) |  |
|  |  | Vitamin A-rich fruits and vegetables | 19 (3.4) |  |
|  |  | Other fruits and vegetables | 499 (89.4) |  |
| 434 |  |  |  |  |

435	Table 4 Distribution of maternal DDS by different variables in Jimma Zone, Southwest Ethiopia

|  | **n (%)** | **n (%)** | **(P-value)** |
| --- | --- | --- | --- |
|  |  |  |  |
|  |  |  |  |
|  |  |  |  |
|  |  |  |  |
|  |  |  |  |
|  |  |  | |
|  |  |  | |
|  |  |  | |
|  |  |  | |
|  |  |  | |
|  |  |  | |
|  |  |  | |
|  |  |  | |
|  |  |  |  |
|  |  |  |  |

# Variables Low DDS Moderate or High DDS *χ2*

Districts

Cash crop-producing)	86 (46.2)	100 (53.8)

Cereal producing)	120 (64.2)	66 (35.5)

Vegetable producing)	93 (50.0)	93 (50.0) Place of residence

0.001**

Rural	220 (59.1)	152 (40.9)	0.000**

Urban	79 (42.5)	107 (57.5)

Age group

15-19	10 (38.5)	16 (61.5)

20-24	61 (42.1)	84 (57.9)

25-29	122 (56.7)	93 (43.3)

30-34	68 (59.1)	47 (40.9)

35-49	38 (66.7)	19 (33.3)

Educational status

0.002**

Informal education	212 (62.7)	126 (37.3)	0.000**

Formal education	87 (39.5)	133 (60.5)

Socio-economic status

Poor	132 (71.0)	54 (29.0)

436

437

Medium	98 (52.7)	88 (47.3)

Rich	69 (37.1)	117 (62.9)

**=Significant at the 0.01 level

0.000**

438	Table 5 The proximate and energy composition of sampled maternal foods in three districts of

| 440 | T=Teff; S=Sorghum; M=Maize; R=Rice; W=Wheat; RDA=Recommended Daily Allowance |
| --- | --- |
| 441 | ^§^(FAO/WHO/UNU, 2004) |
| 442 | $(FAO/WHO/UNU, 1981); Unit for energy it is Kcal/day. |
| 443 |  |

439	Jimma Zone, Southwest Ethiopia from March-May 2014

| Food types | Protein | Fat | Carbohydrate | Fibre | Ash | Energy |
| --- | --- | --- | --- | --- | --- | --- |
|  | g/100g | g/100g | g/100g | g/100g | g/100g | Kcal/100g |
| Lentil sauce | 18.2 | 23.1 | 4.8 | 5.2 | 11.9 | 299.6 |
| Bean sauce | 19.8 | 17.5 | 10 | 5.3 | 11 | 276.5 |
| Pea powder sauce | 16.8 | 19. 8 | 2.2 | 4.3 | 16.9 | 254.2 |
| Kale sauce | 19.6 | 6.5 | 0.9 | 27 | 21.2 | 140.5 |
| *Injera* (T) | 10.1 | 2.6 | 45.6 | 2.5 | 1.5 | 246 |
| *Injera* (T+S) | 10.7 | 2.8 | 43.8 | 2.2 | 1.8 | 243.5 |
| *Injera* (T+M) | 9.6 | 7.5 | 34.8 | 7.6 | 2.6 | 245.1 |
| *Injera* (T+M+S) | 9 | 3.3 | 39.2 | 12.2 | 2 | 222.3 |
| *Injera* (T+S+R) | 9.9 | 5.5 | 8.9 | 8 | 2.1 | 124.5 |
| Unleavened bread (W+M) | 10.8 | 2.1 | 45.8 | 2 | 1.2 | 245.2 |
| Unleavened bread (M) | 7.6 | 3.5 | 42.8 | 2 | 1 | 233.1 |
| Unleavened bread (W) | 11.5 | 3.5 | 33.3 | 2.1 | 1.5 | 210.5 |
| RDA (g/day) § | 65 | 69.1 | 210 | 30 | NA | 2750$ |

444	Table 6 The mineral and anti-nutritional factor content of sampled maternal foods in three

445	districts of Jimma Zone, Southwest Ethiopia

| Food types | Iron | Zinc | Calcium | Phosphorous | Phytate | Tannin |
| --- | --- | --- | --- | --- | --- | --- |
|  | mg/100g | mg/100g | mg/100g | mg/100g | mg/100g | mg/100g |
| Lentil sauce | 20 | 3.4 | 0.2 | 0.3 | 92 | 14.17 |
| Bean sauce | 20.5 | 3.4 | 0.2 | 0.4 | BDL | 27.06 |
| Pea powder sauce | 27.6 | 4.2 | 0.3 | 0.3 | 11.06 | 65.26 |
| Kale sauce | 24.8 | 2.8 | 0.1 | 0.3 | 47.65 | 205.05 |
| *Injera* (T) | 21.8 | 3.4 | 0.3 | 0.3 | 120.45 | BDL |
| *Injera* (T+S) | 39.7 | 3.2 | 0.2 | 0.4 | 145.01 | 81.64 |
| *Injera* (T+M) | 52.5 | 3.1 | 0.3 | 0.3 | 178.89 | 6.64 |
| *Injera* (T+M+S) | 39.4 | 2.7 | 0.2 | 0.4 | 121.61 | 315.85 |
| *Injera* (T+S+R) | 22 | 2.2 | 0.2 | 0.2 | 150.82 | 13.71 |
| Unleavened bread (W+M) | 9.5 | 2.2 | 0.1 | 0.3 | 80.21 | 3.71 |
| Unleavened bread (M) | 12.5 | 2.5 | 0.3 | 0.3 | BDL | 8.36 |
| Unleavened bread (W) | 20.7 | 3.8 | 0.2 | 0.3 | 55.17 | 5.46 |
| RDA (mg/day) | 10 | 12 | 1000 | 1000 | NA | NA |

446	T=Teff; S=Sorghum; M=Maize; R=Rice; W=Wheat; BDL=Below Detectable Level

447

448	Table 7 Adequacy of the maternal foods in nutrients for meeting dietary recommendations

| 449 | P=Protein; Fat=Fat; CHO=Carbohydrate; Fi=Fiber; MC=Moisture Content; Cal=Energy |
| --- | --- |
| 450 | Content; Fe=Iron; Zn=Zinc; Ca=Calcium; P=Phosphorous. T=Teff; S=Sorghum; M=Maize; |
| 451 | R=Rice; W=Wheat |

| **Food types** | **P** | **F** | **CHO** | **Fi** | **Energy** | **Fe** | **Z** | **Ca** | **P** | **MAR** |
| --- | --- | --- | --- | --- | --- | --- | --- | --- | --- | --- |
| Lentil sauce | 0.5 | 0.6 | 0.0 | 0.3 | 0.2 | 3.5 | 0.5 | 0.4 | 0.5 | 0.44 |
| Bean sauce | 0.5 | 0.4 | 0.1 | 0.3 | 0.2 | 3.6 | 0.5 | 0.4 | 0.7 | 0.45 |
| Pea powder sauce | 0.5 | 0.5 | 0.0 | 0.3 | 0.2 | 4.8 | 0.6 | 0.5 | 0.5 | 0.45 |
| Kale sauce | 1.4 | 0.4 | 0.0 | 4.1 | 0.2 | 11.2 | 1.1 | 0.2 | 1.3 | 0.65 |
| *Injera* (T) | 0.9 | 0.2 | 1.2 | 0.5 | 0.5 | 12.0 | 1.5 | 1.8 | 1.9 | 0.78 |
| *Injera* (T+S) | 0.9 | 0.2 | 1.2 | 0.4 | 0.5 | 21.9 | 1.5 | 1.3 | 2.1 | 0.78 |
| *Injera* (T+M) | 0.8 | 0.6 | 0.9 | 1.4 | 0.5 | 28.9 | 1.4 | 1.7 | 1.8 | 0.86 |
| *Injera* (T+M+S) | 0.8 | 0.3 | 1.0 | 2.2 | 0.4 | 21.7 | 1.2 | 1.0 | 2.1 | 0.82 |
| *Injera* (T+S+R) | 0.8 | 0.4 | 0.2 | 1.5 | 0.3 | 12.1 | 1.0 | 1.0 | 0.8 | 0.73 |
| Unleavened bread (W+M) | 0.9 | 0.2 | 1.2 | 0.4 | 0.5 | 5.2 | 1.0 | 0.5 | 1.5 | 0.71 |
| Unleavened bread (M) | 0.6 | 0.3 | 1.1 | 0.4 | 0.5 | 6.9 | 1.2 | 1.5 | 1.5 | 0.75 |
| Unleavened bread (W) | 1.0 | 0.3 | 0.9 | 0.4 | 0.4 | 11.4 | 1.7 | 0.8 | 1.9 | 0.75 |

STROBE checklist

[Click here to access/download](https://www.editorialmanager.com/pone/download.aspx?id=26897612&amp;guid=21be248b-624b-48b9-b24e-9445375a6e3a&amp;scheme=1)

[**Supporting Information** STROBE_checklist_v4_cross-sectional SF.pdf](https://www.editorialmanager.com/pone/download.aspx?id=26897612&amp;guid=21be248b-624b-48b9-b24e-9445375a6e3a&amp;scheme=1)
