# Supplementary material for: Maternal dietary practices, dietary diversity, and nutrient composition of diets of lactating mothers in Jimma Zone, Southwest Ethiopia
Source: PLoS One. 2021 Jul 12;16(7):e0254259. doi: 10.1371/journal.pone.0254259 (PMC8274933; doi:10.1371/journal.pone.0254259)
Supplement: Attachment — Submitted filename: Response to Reviewers.docx [file pone.0254259.s005.docx]

# Journal Requirements:

When submitting your revision, we need you to address these additional requirements.

**Journal requirement 1:**

1. Please ensure that your manuscript meets PLOS ONE's style requirements, including those for file naming. The PLOS ONE style templates can be found at https://journals.plos.org/plosone/s/file?id=wjVg/PLOSOne_formatting_sample_main_body.pdf and https://journals.plos.org/plosone/s/file?id=ba62/PLOSOne_formatting_sample_title_authors_affiliations.pdf

**Authors' response 1:**

Both the title page and the main body of the manuscript were formatted according to PLOS ONE's style requirements. See the revised manuscript for details.

**Journal requirement 2:**

2. We note that the grant information you provided in the ‘Funding Information’ and ‘Financial Disclosure’ sections do not match. When you resubmit, please ensure that you provide the correct grant numbers for the awards you received for your study in the ‘Funding Information’ section.

**Authors' response 2:**

Lines 428-430: The funding information was updated as follows:

“Funding information

This research was funded by the Bundesministerium für Bildung und Forschung, Grant/Award Number: 031A247A.”

**Journal requirement 3:**

3. In your Data Availability statement, you have not specified where the minimal data set underlying the results described in your manuscript can be found. PLOS defines a study's minimal data set as the underlying data used to reach the conclusions drawn in the manuscript and any additional data required to replicate the reported study findings in their entirety. All PLOS journals require that the minimal data set be made fully available. For more information about our data policy, please see http://journals.plos.org/plosone/s/data-availability.

Upon re-submitting your revised manuscript, please upload your study’s minimal underlying data set as either Supporting Information files or to a stable, public repository and include the relevant URLs, DOIs, or accession numbers within your revised cover letter. For a list of acceptable repositories, please see http://journals.plos.org/plosone/s/data-availability#loc-recommended-repositories. Any potentially identifying patient information must be fully anonymized.

Important: If there are ethical or legal restrictions to sharing your data publicly, please explain these restrictions in detail. Please see our guidelines for more information on what we consider unacceptable restrictions to publicly sharing data: http://journals.plos.org/plosone/s/data-availability#loc-unacceptable-data-access-restrictions. Note that it is not acceptable for the authors to be the sole named individuals responsible for ensuring data access.

We will update your Data Availability statement to reflect the information you provide in your cover letter.

**Authors' response 3:**

We have uploaded our study’s minimal underlying data set as a stable, public repository and included the relevant DOI (https://doi.org/10.5061/dryad.nk98sf7sr) within our revised cover letter.

**Journal requirement 4:**

4. We noted in your submission details that a portion of your manuscript may have been presented or published elsewhere.

"This work has been presented at a conference Tropentag 2016 (https://www.tropentag.de/2016/abstracts/full/100.pdf)."

Please clarify whether this [conference proceeding or publication] was peer-reviewed and formally published. If this work was previously peer-reviewed and published, in the cover letter please provide the reason that this work does not constitute dual publication and should be included in the current manuscript.

**Authors' response 4:**

Only the abstract part, which is submitted to the conference, has passed through a peer-review process to become acceptable for oral presentation at the conference. The 4 page summary did not pass through a peer-review process and the authors’ believe it does not constitute dual publication.

**Journal requirement 5:**

5. Please include captions for your Supporting Information files at the end of your manuscript, and update any in-text citations to match accordingly. Please see our Supporting Information guidelines for more information: http://journals.plos.org/plosone/s/supporting-information.

[Note: HTML markup is below. Please do not edit.]

**Authors' response 5:**

Line 583-585: The following caption is added to describe the supporting information files:

“Supporting information 1: Minimal underlying data set on maternal dietary practices, dietary diversity, and nutrient composition of diets of lactating mothers in Jimma zone, Southwest Ethiopia”

Line 586: The following caption is added to describe the supporting information files:

“Supporting information 2: type of stews consumed and ingredients of the stews”

The Supporting Information data set file is not cited in-text.

Reviewers' comments:

Reviewer's Responses to Questions

Comments to the Author

1. Is the manuscript technically sound, and do the data support the conclusions?

The manuscript must describe a technically sound piece of scientific research with data that supports the conclusions. Experiments must have been conducted rigorously, with appropriate controls, replication, and sample sizes. The conclusions must be drawn appropriately based on the data presented.

Reviewer #1: Yes

Reviewer #2: Yes

Reviewer #3: No

Reviewer #4: Partly

2. Has the statistical analysis been performed appropriately and rigorously?

Reviewer #1: Yes

Reviewer #2: Yes

Reviewer #3: No

Reviewer #4: No

3. Have the authors made all data underlying the findings in their manuscript fully available?

The PLOS Data policy requires authors to make all data underlying the findings described in their manuscript fully available without restriction, with rare exception (please refer to the Data Availability Statement in the manuscript PDF file). The data should be provided as part of the manuscript or its supporting information, or deposited to a public repository. For example, in addition to summary statistics, the data points behind means, medians and variance measures should be available. If there are restrictions on publicly sharing data—e.g. participant privacy or use of data from a third party—those must be specified.

Reviewer #1: Yes

Reviewer #2: No

Reviewer #3: No

Reviewer #4: No

4. Is the manuscript presented in an intelligible fashion and written in standard English?

PLOS ONE does not copyedit accepted manuscripts, so the language in submitted articles must be clear, correct, and unambiguous. Any typographical or grammatical errors should be corrected at revision, so please note any specific errors here.

Reviewer #1: Yes

Reviewer #2: No

Reviewer #3: No

Reviewer #4: No

5. Review Comments to the Author

Please use the space provided to explain your answers to the questions above. You may also include additional comments for the author, including concerns about dual publication, research ethics, or publication ethics. (Please upload your review as an attachment if it exceeds 20,000 characters)

# Reviewer #1:

**Reviewer 1 comment 1:**

Concerns that the data was collected about 6 years ago ie 2014

**Authors' response 1:**

That is true.

**Reviewer 1 comment 2:**

There are a few grammatical errors that should be reviewed in the manuscript and corrected - need to improve in certain parts on the English.

**Authors' response 2:**

Language edition is done using a premium subscription of Grammarly (a digital writing assistance tool based on artificial intelligence and natural language processing). See revised manuscript.

**Reviewer 1 comment 3:**

There is reference in the abstract to "little stores" - I propose this be altered to minimal stores

**Authors' response 3:**

Line 24: Corrected as suggested by the reviewer.

**Reviewer 1 comment 4:**

Would have been good to make reference to the overall maternal mortality rate in Ethiopia including causes - and if any are related to nutritional deficiencies. More data on the general health of the subjects in the study would help

**Authors' response 4:**

Lines 68-70: The following sentence is added to show the relation between nutritional deficiencies and maternal mortality.

“Ethiopia experiences one of the highest incidences of child and maternal nutritional deficiencies which contribute to increased morbidity and mortality (Central Statistical Agency [Ethiopia] & ICF International, 2012).”

**Reviewer 1 comment 5:**

In mentioning the vulnerability of mothers to undernutrition in this context, would help to also mention the possibility of intergenerational cycles of malnutrition

**Authors' response 5:**

Lines 70-72: The following sentence is added:

“A malnourished mother will give birth to a low birth weight baby; the low birth weight baby will grow as a malnourished child, then to a malnourished teenager, then to a malnourished pregnant woman, and so the cycle continues (10).”

Lines 471-473: The following reference was added to the reference list:

“Ramakrishnan U, Martorell R, Schroeder DG, Flores R. Role of intergenerational effects on linear growth. In: Journal of Nutrition [Internet]. American Institute of Nutrition; 1999 [cited 2021 Mar 25]. Available from: https://pubmed.ncbi.nlm.nih.gov/10064328/”

**Reviewer 1 comment 6:**

Whilst most of the study participants were Muslim, I wondered the extent to which those who were christian were Orthodox Christians and could have still adhered to fasting regimes even during lactation - a comment on this aspect would be useful

**Authors' response 6:**

Out of 558 participants, there were 42 Christian participants in the study out of which 33 (78.5%) were Orthodox Christians. However, there was neither fasting nor feasting when we undergone the survey.

**Reviewer 1 comment 7:**

In order to understand the food security, income and poverty dimension, it would help to know how many of the mothers resided in PSNP woredas/ kebelles and therefore may have been enrolled in this social protection scheme which could have influenced dietary diversity.

**Authors' response 7:**

Only chronically food insecure districts (woredas) are enrolled in PSNP. None of our study districts were in this social protection scheme.

**Reviewer 1 comment 8:**

Whilst there is mention of consumption of iodised salt - at the time the survey data was collected the quality of iodised salt was very poor - with minimal levels

**Authors' response 8:**

What we meant by the sentences in lines 312-315 is the consumption of iodized salt in the study area is very low compared to other areas of the country where media coverage (radio and television) coverage is wider.

**Reviewer 1 comment 9:**

Under limitations I propose to include the fact that the dietary data alone does not refer to bioavailabilty of the nutrients which are influenced by the overall health of the mothers

**Authors' response 9:**

Lines 400-402: The following sentence was added as suggested by the reviewer:

“Additionally, the dietary data alone does not refer to bioavailability of the nutrients, which are influenced by the overall health of the mothers.”

# Reviewer #2:

Thank you for the opportunity to review this paper. My comments are as follows:

**Reviewer 2 comment 1:**

There are some minor issues with English throughout that need to be addressed. For example:

Line 169 "Majority" should be "The majority.."

**Authors' response 1:**

Line 191: “Majority…” is changed to “The majority…”

Line 200: “Majority…” is changed to “The majority…”

The English was edited by a premium version of Grammarly.

**Reviewer 2 comment 2:**

Line 172 "When it comes to the practice.." should be "In practice.."

**Authors' response 2:**

Lines 203-204: Modified as suggested by the reviewer

**Reviewer 2 comment 3:**

Line 210 "played its role for this" should be "played a role in this"

**Authors' response 3:**

Line 297: The phrase “fermentation is believed to have played its role for this” is deleted as it is out of context.

**Reviewer 2 comment 4:**

Line 214 "Majority" should be "The majority.."

**Authors' response 4:**

Line 306: “Majority…” is changed to “The majority…”

**Reviewer 2 comment 5:**

Abstract: Line 17 "little stores" should perhaps be "low stores"

**Authors' response 5:**

Line 24: "little stores" is changed to "minimal stores"

**Reviewer 2 comment 6:**

Line 27 DDS should be defined

**Authors' response 6:**

Line 36: “DDS” was changed to “dietary diversity score (DDS)”

**Reviewer 2 comment 7:**

Line 40 should be "nutrition education program"

**Authors' response 7:**

Line 53: Corrected as suggested by the reviewer

**Reviewer 2 comment 8:**

Abstract is otherwise appropriate.

**Authors' response 8:**

Ok

**Reviewer 2 comment 9:**

Introduction: Appropriate.

**Authors' response 9:**

Ok

**Reviewer 2 comment 10:**

Methods: Maternal dietary practices section, line 109, what is meant by semi-structured questionnaires? Questionnaires are generally structured in nature - do the authors mean semi-structured interviews?

**Authors' response 10:**

Line 130: “questionnaires” is changed to “interviews”

**Reviewer 2 comment 11:**

Line 115 to calculate DDS was 1 point awarded for the consumption of each food group with a total possible score being 9? This should be explained in the text.

**Authors' response 11:**

Lines 142 - 143: The following sentence is added as suggested by the reviewer:

“One point was awarded for the consumption of each food group with a total possible score being 10.”

NB. As suggested by another reviewer, the FAO & FHI 360, 2016 methodology, which is based on 10 food groups, was used to recalculate the dietary diversity of the women.

**Reviewer 2 comment 12:**

Line 116-117 "greater than 3.4 mean food groups" This word is not correct and should be something like "a mean (or average) number of food groups greater than 3.4".

**Authors' response 12:**

Line 144-: corrected as suggested by the reviewer:

“mean number of food groups greater than 5”

NB. According to the FAO & FHI 360, 2016 methodology, which is based on 10 food groups, the cut-off is ≥5 food groups.

**Reviewer 2 comment 13:**

Line 117 "respondents with <3.4 mean food groups" is also not correct and should be "respondents with mean of less than 3.4 food groups..."

**Authors' response 13:**

Line 145: corrected as suggested by the reviewer:

“respondents with mean of less than 3.4 food groups”

**Reviewer 2 comment 14:**

Line 121 Please clarify as to what composite samples are. Are they combined samples or just samples of each food individually?

**Authors' response 14:**

Line 149-150: The following phrase was added to explain what composite samples mean.

“(a mixture of individual samples)”

**Reviewer 2 comment 15:**

Line 128 "gram of portion per day" is not clear. Should is just be "grams per day"?

**Authors' response 15:**

Line 156: corrected as follows.

“recommended portion per day”

Line 158: corrected as follows.

“recommended portion per day”

**Reviewer 2 comment 16:**

Were t-test considered to compare means between groups?

**Authors' response 16:**

We did not understand this comment. We did not conduct a t-test to compare means between groups.

**Reviewer 2 comment 17:**

Results:

Line 185 What is "organ" meat?

**Authors' response 17:**

Line 138; Line 221: organ meat is deleted from the manuscript as it is not included in the FAO & FHI 360 (2016)´s ten food groups.

**Reviewer 2 comment 18:**

Line 190-191 sentence beginning "This study.." is not presenting results and should be removed.

**Authors' response 18:**

Line 231-232: The following sentence is deleted:

“This study showed the distribution of dietary diversity over different influencing factors.”

**Reviewer 2 comment 19:**

Discussion: Line 215-216 is not clear and needs to be reworked.

**Authors' response 19:**

Lines 306-309: The sentence was revised in order to improve clarity.

“…food intake habit (quantity, quality and diversity) during lactation, which is in contrast to the recommendations in the essential nutrition actions…”

**Reviewer 2 comment 20:**

Line 222 is not clear.

**Authors' response 20:**

Line 314: the following phrase is added to explain what media refers to.

“(FM radios and television)”

**Reviewer 2 comment 21:**

Line 232 could the authors please define what they mean by cash crop

**Authors' response 21:**

Line 324: The following word is added to explain what cash crop meant in this study’s context:

“(coffee)”

**Reviewer 2 comment 22:**

Line 242, incorrect reference format for Ruel

**Authors' response 22:**

Line 334: “(Ruel, 2003)” changed to “Ruel (2003)”

**Reviewer 2 comment 23:**

Paragraph 240-244 is not clear e.g. what is "placed in the separate residence"?

**Authors' response 23:**

Line 332-333: "placed in the separate residence" is replaced with “living in urban and rural residences”

**Reviewer 2 comment 24:**

Line 245 incorrect reference format for Ajani. Again, this paragraph is not clear.

**Authors' response 24:**

Line 337: “(Ajani, 2010)” is changed to “Ajani (2010)”

Line 337-340: The paragraph is revised as follows:

“According to Ajani (2010) educational status and household wealth were interrelated and had a positive association with dietary diversity. Higher education attainment is likely to be associated with a higher income and thereby money to buy different food commodity for household food preparation.”

**Reviewer 2 comment 25:**

Line 253, could the authors please describe what injera is. For example, is it like bread?

**Authors' response 25:**

Line 349-350: The following phrase is added to describe what Injera is.

“(Ethiopian traditional cereal-based pancake, prepared from fermented cereal sour dough)”

**Reviewer 2 comment 26:**

Line 257 please indicate what is meant by ash.

**Authors' response 26:**

Lines 354-355: The following phrase is added to explain what ash content means:

“a measure of the total amount of minerals present within a food”

**Reviewer 2 comment 27:**

Line 266 has Ca been previously defined (it is assumed this is calcium?)

**Authors' response 27:**

Line 363: corrected as follows:

“calcium (Ca)”

**Reviewer 2 comment 28:**

Line 251 - kale sauce has been highlighted specifically in the discussion but has not been described previously. It would be of benefit to describe how kale sauce is used, or what part it plays in the diet prior to this.

**Authors' response 28:**

Line 345-348: the following sentence is added to describe how kale sauce is used:

“Kale sauce is prepared by cooking onion and oil 4 to 5 minutes and adding chopped and cooked-until-soft kale, and stirring once or twice, for 10 minutes. During our survey we have observed that most households add protein rich ingredients like beans when making kale sauce.”

**Reviewer 2 comment 29:**

Limitations - appropriate although how representative is this study of the rest of Ethiopia?

**Authors' response 29:**

Lines 399-400: The following limitation statement is added as suggested by the reviewer.

“Ethiopia is a very diverse country in terms of food production and consumption culture, and this study may not be representative of the rest of the country.”

**Reviewer 2 comment 30:**

Strengths - I am unclear how this is a strength. This is an outcome from the study but the methodology undertaken to achieve this is the strength.

**Authors' response 30:**

Lines 404-405: The sentence was rewritten as follows:

“An official food analysis methodology was employed to analyze the composition of undocumented maternal diets.”

**Reviewer 2 comment 31:**

Conclusions:

Line 307-308 this sentence is not clear and should be reworked.

**Authors' response 31:**

Lines 410-412: The sentence is revised as follows:

“Kale sauce, which is traditionally popular in South and Southwest part of rural Ethiopia, tend to provide relatively healthy macro and micronutrients to the lactating mothers.”

**Reviewer 2 comment 32:**

Table 4 - why was 0.01 used as the p value when .05 is the standard and this is stated in line 147.

**Authors' response 32:**

Line 246: corrected as suggested by the reviewer.

**Reviewer 2 comment 33:**

Other tables appropriate although only descriptive in nature

**Authors' response 33:**

Lines 257-263: A new table is added:

“Table 5. Multivariable logistic regression model predicting the likelihood of having Minimum Dietary diversity (MDD-W) among women in Jimma Zone, Southwest Ethiopia”

# Reviewer #3:

**Reviewer 3 comment 1:**

While the topic of the study has public health importance, measurement approaches, the tools and the data analysis is not scientifically sounding. Despite the result section and conclusion attempted to note the variables associated with low dietary diversity there is no a strong method of data analysis to test the hypothesis. The study used simple chi-square which simply allows us to see the presence or absence of any association between two variables at a time. This does not help us to know which variable is cause and which is an effect? In what magnitude? In what direction (favoring or not).

**Authors' response 1:**

Line 174-175:

“Chi-square test was used to see the association between dietary diversity and socio-economic and demographic variables.”

was changed to

“Bivariate and multivariate logistic regression statistical analysis was carried out to determine the factors associated with diet diversity in lactating mothers.”

Lines 249-271: The following results of multivariate logistic regression were added:

“On multivariable logistic regression analyses presented in Table 5, after adjusting for different variables, meeting Minimum dietary diversity (MDDS) of women was positively associated with agricultural production diversity(P=0.001) and grade of the women(P=0.04). For a unit increase in agricultural production diversity of the household, the likelihood of achieving minimum dietary diversity of women was 1.4 times higher (AOR: 1.394, 95%CI: 1.142, 1.701). Similar, for one grade increase in the educational status of women the odds of achieving minimum, dietary diversity was 1.5 times higher( AOR: 1.485, 95%CI: 1.018, 2.168).

Table 5. Multivariable logistic regression model predicting the likelihood of having Minimum Dietary diversity (MDD-W) among women in Jimma Zone, Southwest Ethiopia

| Model | B | P | AOR | 95% C.I. |
| --- | --- | --- | --- | --- |

| Lower | Upper |
| --- | --- |

| District |  |  |  |  |  |
| --- | --- | --- | --- | --- | --- |

| Manna | -0.211 | 0.599 | 0.810 | 0.369 | 1.779 |
| --- | --- | --- | --- | --- | --- |

| Omo-nada | -1.444 | 0.003 | 0.236 | 0.090 | 0.616 |
| --- | --- | --- | --- | --- | --- |

| Dedo |  |  | 1.000 |  |  |
| --- | --- | --- | --- | --- | --- |

| Food Production diversity | 0.332 | 0.001 | 1.394 | 1.142 | 1.701 |
| --- | --- | --- | --- | --- | --- |

| Grade of the women | 0.396 | 0.040 | 1.485 | 1.018 | 2.168 |
| --- | --- | --- | --- | --- | --- |

| Wealth Tertitle |  |  |  |  |  |
| --- | --- | --- | --- | --- | --- |

| Poor | -0.732 | 0.154 | 0.481 | 0.176 | 1.317 |
| --- | --- | --- | --- | --- | --- |

| Medium | -1.013 | 0.062 | 0.363 | 0.125 | 1.052 |
| --- | --- | --- | --- | --- | --- |

| Rich |  |  | 1.000 |  |  |
| --- | --- | --- | --- | --- | --- |

| Age of the woman(yrs) | -0.065 | 0.135 | 0.937 | 0.861 | 1.020 |
| --- | --- | --- | --- | --- | --- |

| Household has alternative income source | -0.175 | 0.639 | 0.839 | 0.404 | 1.744 |
| --- | --- | --- | --- | --- | --- |

| Place of residence, |  |  |  |  |  |
| --- | --- | --- | --- | --- | --- |

| Rural | -1.263 | 0.019 | 0.283 | 0.099 | 0.810 |
| --- | --- | --- | --- | --- | --- |

| Urban |  |  | 1.00 |  |  |
| --- | --- | --- | --- | --- | --- |

| Educational status of the husband | 0.894 | 0.085 | 2.446 | 0.883 | 6.776 |
| --- | --- | --- | --- | --- | --- |

| Household size | 0.150 | 0.171 | 1.162 | 0.937 | 1.441 |
| --- | --- | --- | --- | --- | --- |

*Production diversity: a score generated by summing the number of animal and plant source food that the household reported to produce.*

*Maximum Standard error: 0.543, Hosmer Lemeshow Test for Model Fitness (P=0.052).*

*AOR: Adjusted Odds Ratio.*

*CI: Confidence Interval.*

Conversely, district of the study (P=0.003) and place of residence (P= 0.019 ) were negatively associated with meeting minimum dietary diversity. Women living in Omo-Nada District were 86% less likely to meet the minimum dietary diversity as compared to this who live in Dedo District( AOR: 0.236, 95%CI” 0.090, 0.616). Likewise, women who live in the rural areas were 72% less likely to meet the minimum dietary diversity as compared to those who live in the urban areas (AOR: 0.283, 95%CI: 0.099, 0.810).”

**Reviewer 3 comment 2:**

Another concern is, construction of survey questionnaire has not been clearly stated and its reliability and validity are not presented. For example, measuring socioeconomic class, educational status etc was not clear.

**Authors' response 2:**

Line 29-30

“pre-tested and”

Line 121-124: the following paragraph was added to describe how the wealth index was calculated:

**“Socio-economic information**

A wealth index was determined using principal component analyses based on data on assets encompassing land for agriculture, production of crops, ownership of animals and properties (Garenne & Hohmann-Garenne, 2003). The index was rank divided into tertiles and used for further analyses.”

Line 492-493: The following reference is added to the reference list:

Garenne M, Hohmann-Garenne S (2003) A wealth index to screen high-risk families: Application to Morocco. J Heal Popul Nutr 21:235–242

**Reviewer 3 comment 3:**

Including different agroecological areas in this study could be taken as one of the strengths. However, it is true that dietary diversity could vary seasonally, and the study has not mentioned the season(s) when the study was undertaken.

**Authors' response 3:**

Line 107: The following phrase is added to indicate the season of the study:

“post-harvest season which is dry”

# Reviewer #4:

**Reviewer 4 comment 1:**

Consideration of the nutrient needs of lactating women is an important topic, particularly as longer periods of lactation are being recommended.

**Authors' response 1:**

Ok

**Reviewer 4 comment 2:**

An important reference that the authors will want to consider is: FAO and FHI 360. 2016. Minimum Dietary Diversity for Women: a Guide for Measurement. Rome: FAO. The MDD-W has been utilized and validated by several groups. The MDD-W presented in this publication outlines 10 food groups (which are not the same as those specified in this manuscript. And it specifies a cut-off of ≥5 food groups.

**Authors' response 2:**

We accepted the reviewer's comments and corrected the dietary diversity related information in the manuscript accordingly.

Lines 133-146:

“A single 24-h dietary recall was used to obtain data on dietary diversity. Dietary diversity was assessed with the ten MDD-W food groups namely (1) Grains, white roots and tubers, and plantains, (2) Pulses (beans, peas and lentils), (3) Nuts and seeds (4) Dairy, (5) Meat, poultry and fish, (6) Eggs, (7) Dark green leafy vegetables, (8) Other vitamin A-rich fruits and vegetables, (9) Other vegetables and (10) Other fruits. One point was awarded for the consumption of each food group with a total possible score being 10. Minimum dietary diversity was found to be achieved when a mother consumed mean number of food groups greater than 5 per day and respondents with mean of less than 5 food groups were considered as not achieving the minimum dietary diversity (FAO & FHI 360, 2016).”

Lines 209-263: The dietary diversity results were revised as follows:

Lines 226-227: Table 3 was revised following the FAO & FHI 360, 2016 methodology:

Table 3 Dietary diversity score and percentage of women consuming each of the 9 food groups

|  | | DDS | |
| --- | --- | --- | --- |
| Mean | | 3.73 | |
| Median | | 4.0 | |
| SD | | 1.03 | |
| Minimum | | 0 | |
| Maximum | | 8 | |
| Minimum Dietary Diversity for Women of Reproductive Age | | N (%) | |
| <5 food groups | 447 (80.1) | |  |
| ≥5 food groups | 111 (19.9) | |  |
| Consumption | | N (%) | |
| Grains, white roots and tubers, and plantains | 553 (99.1) | |  |
| Pulses (beans, peas and lentils) | 431 (77.2) | |  |
| Nuts and seeds | 13 (2.3) | |  |
| Dairy | 75 (13.4) | |  |
| Meat, poultry and Fish | 18 (3.2) | |  |
| Eggs | 7 (1.3) | |  |
| Dark green leafy vegetables | 299 (53.6) | |  |
| Other vitamin A-rich vegetables and fruits | 58 (10.4) | |  |
| Other vegetables | 499 (89.4) | |  |
| Other fruits | 129 (23.1) | |  |

Lines 230-238: The presence or absence of any association between MDD and socio-economic and demographic variables at a time was revised according to the FAO & FHI 360, 2016 methodology.

“Table 4 shows the association between socio-demographic variables and maternal dietary diversity. Accordingly, dietary diversity score was significantly (P<0.01) affected by districts, residence area, ethnicity, whether the woman is currently attending education or not, husband’s grade and the socio-economic situation of the household. Oromo mothers who live in cereal producing districts, who are not currently attending education, living in poor households and whose husband attended lower grade education were more likely not to achieve MDD as compared with their respective counterparts.”

Lines 244-246: Table 4 was revised accordingly:

Table 4 Distribution of maternal dietary diversity by different variables in Jimma Zone, Southwest Ethiopia

| Variables | Did not achieve MDD | Achieved MDD |  | |
| --- | --- | --- | --- | --- |
|  | **n (%)** | **n (%)** | **P** | |
| Districts |  |  |  | |
| Cash crop | 146 (78.5) | 40 (21.5) | 0.019* | |
| Cereal | 161 (86.6) | 25 (13.4) |  |  |
| Vegetable | 140 (75.3) | 46 (24.7) |  |  |
| Ethnicity |  |  |  | |
| Oromo | 397 (81.7) | 89 (18.3) | 0.005* | |
| Amhara | 16 (80.0) | 4 (20.0) |  |  |
| Guraghe | 6 (75.0) | 2 (25.0) |  |  |
| Tigre | 2 (66.7) | 1 (33.3) |  |  |
| Yem | 6 (40.0) | 9 (60.0) |  |  |
| Other | 20 (76.9) | 6 (23.1) |  |  |
| Educational status of the woman |  |  |  | |
| 1 to 4 | 76 (78.4) | 21 (21.6) | 0.134 | |
| 5 to 8 | 65 (76.5) | 20 (23.5) |  |  |
| 9 to 10 | 19 (65.5) | 10 (34.5) |  | |
| Preparatory/TVET | 4 (44.4) | 5 (55.6) |  | |
| Diploma | 1 (50.0) | 1 (50.0) |  | |
| Currently attending education |  |  |  | |
| No | 446 (80.5) | 108 (19.5) | 0.006* | |
| Yes | 1 (25.0) | 3 (75.0) |  |  |
| Husband's grade |  |  |  | |
| 1 to 4 | 73 (83.9) | 14 (16.1) | 0.004* | |
| 5 to 8 | 132 (78.6) | 36 (21.4) |  |  |
| 9 to 10 | 26 (57.8) | 19 (42.2) |  |  |
| Preparatory/TVET | 17 (89.5) | 2 (10.5) |  |  |
| diploma | 2 (50) | 2 (50) |  |  |
| Place of residence |  |  |  | |
| Rural | 297 (79.8) | 75 (20.2) | 0.822* | |
| Urban | 150 (80.6) | 36 (19.4) |  |  |
| Socio-economic status | | | | |
| Poor | 155 (83.3) | 31 (16.7) | | 0.046* |
| Medium | 154 (82.8) | 32 (17.2) | |  |
| Rich | 138 (74.2) | 111 (19.9) | |  |

*=Significant at the 0.05 level

Line 496-498: The following reference was added to the list:

“FAO, FHI 360. Minimum Dietary Diversity for Women: A Guide for Measurement [Internet]. Rome: FAO; 2016 Feb [cited 2021 Mar 15]. Available from: http://www.fao.org/documents/card/en/c/cb3434en”

**Reviewer 4 comment 3:**

There are several inconsistencies in the manuscript that the authors must address. Some, but not all, of these inconsistencies are included in the following points:

L. 22-23 says the average MDD-W was computed from two 24-h recalls, while L. 112 says a single 24-h dietary recall….

**Authors' response 3:**

Line 30-31: The reviewer is correct. The sentence was corrected accordingly.

“Minimum diet diversity (MDD-W) was computed from a single 24-h recall.”

**Reviewer 4 comment 4:**

The nine food groups listed on L. 113 -115 are not the same as the FAO publication nor are they identical to the food groups listed in Table 3 of this manuscript.

**Authors' response 4:**

Line 134-137: The food groups are revised according to FAO & FHI 360 (201

).

“(1) Grains, white roots and tubers, and plantains, (2) Pulses (beans, peas and lentils), (3) Nuts and seeds (4) Dairy, (5) Meat, poultry and fish, (6) Eggs, (7) Dark green leafy vegetables, (8) Other vitamin A-rich fruits and vegetables, (9) Other vegetables and (10) Other fruits”

Line 226-227: Table 3 is revised according to FAO & FHI 360 (2016).

**Reviewer 4 comment 5:**

L. 88-89 says kebeles were sampled from the three selected districts by lottery method. If only by lottery, there could have been 0 to 3 urban kebeles selected from one district. Please clarify the sampling procedure.

**Authors' response 5:**

Lines 100-104: The sampling method of the kebeles is revised as follows:

“The total kebeles in each woreda were initially stratified into rural and urban areas. Then, three (two rural and one urban) were selected from the existing kebeles based on Probability Proportional to Size (PPS) sampling technique. A total of six rural and three urban kebeles were sampled from the three districts.”

**Reviewer 4 comment 6:**

L. 127. The document from the Public Health Department of Georgia and World Health Organization (2015) seems to be a report for the general public about healthy eating. Are the recommended grams of portion per day given as dry weight or wet weight. How were these portion sizes adjusted for the calculations in this paper?

**Authors' response 6:**

We acknowledged, as a limitation of our study that the Public Health Department of Georgia and World Health Organization (2015) report is for the general public about healthy eating and may not directly fit to lactating mothers. The grams of portion per day presented in the Georgia report are given on wet basis. While our food composition results are given on dry weight basis. Because nutrients are found in the dry matter portion of foods, the physical quantity of nutrients will NOT change when water is added or removed.

**Reviewer 4 comment 7:**

L. 135 mentions the FAO/WHO RNI intakes for mothers. L. 139 specifies division by the RDA. The RNI and the RDA typically are not the same. RDA appears also on Table 5&6.

**Authors' response 7:**

The definition of RNI used in FAO/WHO report is equivalent to that of recommended dietary allowance (RDA) as used by the Food and Nutrition Board of the US National Academy of Sciences^#^.

^#^Food and Nutrition Board, Institute of Medicine. 1997. Dietary Reference Intakes: Washington, DC, National Academy Press.

**Reviewer 4 comment 8:**

L. 180. Does the Dietary score of zero represent a woman who was fasting??

**Authors' response 8:**

We suspect a recall bias. There was neither fasting nor feasting when we undergone the survey.

**Reviewer 4 comment 9:**

L. 210 Fermentation is very likely to increase the bioavailability of Zn, Fe, and perhaps Ca, but usual laboratory analyses and food composition tables show total mineral concentration not bioavailable mineral concentrations.

**Authors' response 9:**

Line 297: deleted the following phrase as does not fit to the context.

“and fermentation is believed to have played a role in this”

**Reviewer 4 comment 10:**

L. 222. What is meant by different media?

**Authors' response 10:**

Line 314: the following phrase is added to explain what media refers to.

“(FM radios, television)”

**Reviewer 4 comment 11:**

While it is clear that diet diversity was poor and food intakes were inadequate, it is not always clear which differences among the three areas were significantly different from each other. On Table 4, overall differences are shown between “low” and “moderate or high” by chi-square. In L. 224-235 which of the differences described are significantly different when there are more than two categories for a variable?

**Authors' response 11:**

Line 249-271: The following multivariate analysis result is added to the revised manuscript.

“On multivariable logistic regression analyses presented in Table 5, after adjusting for different variables, meeting Minimum dietary diversity (MDDS) of women was positively associated with agricultural production diversity(P=0.001) and grade of the women(P=0.04). For a unit increase in agricultural production diversity of the household, the likelihood of achieving minimum dietary diversity of women was 1.4 times higher (AOR: 1.394, 95%CI: 1.142, 1.701). Similar, for one grade increase in the educational status of women the odds of achieving minimum, dietary diversity was 1.5 times higher( AOR: 1.485, 95%CI: 1.018, 2.168).

Table 5. Multivariable logistic regression model predicting the likelihood of having Minimum Dietary diversity (MDD-W) among women in Jimma Zone, Southwest Ethiopia

| Model | B | P | AOR | 95% C.I. | |
| --- | --- | --- | --- | --- | --- |
|  |  |  |  | Lower | Upper |
| District |  |  |  |  |  |
| Manna | -0.211 | 0.599 | 0.810 | 0.369 | 1.779 |
| Omo-nada | -1.444 | 0.003 | 0.236 | 0.090 | 0.616 |
| Dedo |  |  | 1.000 |  |  |
| Food Production diversity | 0.332 | 0.001 | 1.394 | 1.142 | 1.701 |
| Grade of the women | 0.396 | 0.040 | 1.485 | 1.018 | 2.168 |
| Wealth Tertitle |  |  |  |  |  |
| Poor | -0.732 | 0.154 | 0.481 | 0.176 | 1.317 |
| Medium | -1.013 | 0.062 | 0.363 | 0.125 | 1.052 |
| Rich |  |  | 1.000 |  |  |
| Age of the woman(yrs) | -0.065 | 0.135 | 0.937 | 0.861 | 1.020 |
| Household has alternative income source | -0.175 | 0.639 | 0.839 | 0.404 | 1.744 |
| Place of residence, |  |  |  |  |  |
| Rural | -1.263 | 0.019 | 0.283 | 0.099 | 0.810 |
| Urban |  |  | 1.00 |  |  |
| Educational status of the husband | 0.894 | 0.085 | 2.446 | 0.883 | 6.776 |
| Household size | 0.150 | 0.171 | 1.162 | 0.937 | 1.441 |

*Production diversity: a score generated by summing the number of animal and plant source food that the household reported to produce.*

*Maximum Standard error: 0.543, Hosmer Lemeshow Test for Model Fitness (P=0.052).*

*AOR: Adjusted Odds Ratio.*

*CI: Confidence Interval.*

Conversely, district of the study (P=0.003) and place of residence (P= 0.019) were negatively associated with meeting minimum dietary diversity. Women living in Omo-Nada District were 86% less likely to meet the minimum dietary diversity as compared to this who live in Dedo District( AOR: 0.236, 95%CI” 0.090, 0.616). Likewise, women who live in the rural areas were 72% less likely to meet the minimum dietary diversity as compared to those who live in the urban areas (AOR: 0.283, 95%CI: 0.099, 0.810).”

**Reviewer 4 comment 12:**

A supplementary table is needed to describe the ingredients (recipe) in lentil sauce, bean sauce, pea powder sauce and kale sauce. Particularly for a sauce based on kale, it is hard to imagine a protein concentration of 19.6 g/100 g.

**Authors' response 12:**

A supplementary file which indicates the type of stews consumed and ingredients of the stews is included. See Supporting information 2.spv.

Line 586: The following caption is added to describe the supporting information files:

“Supporting information 2: Type of stews consumed and ingredients of the stews”

Line 347-348: The following sentence is added as a possible explanation for the high protein content recorded for kale sauce.

“During our survey we have observed that most households add protein rich ingredients like beans when making kale sauce.”

**Reviewer 4 comment 13:**

There has been work published on phytate and bioavailability (Food & Nutrition Bulletin, 2010) that the authors might find useful.

**Authors' response 13:**

Line 370: The following word is added:

“(e.g. dephytinization)”

Lines 371-373: the following text was added from the recommended paper.

“combined with enrichment with animal-source foods and/or fortification with appropriate levels and forms of mineral fortificants (Gibson et al., 2010)”

Lines 541-545: The following reference is added to the reference list.

“Gibson, R. S., Bailey, K. B., Gibbs, M., & Ferguson, E. L. (2010). A review of phytate, iron, zinc, and calcium concentrations in plant-based complementary foods used in low-income countries and implications for bioavailability. In *Food and Nutrition Bulletin* (Vol. 31, Issue 2 SUPPL.). United Nations University Press. https://doi.org/10.1177/15648265100312s206”

6. PLOS authors have the option to publish the peer review history of their article (what does this mean?). If published, this will include your full peer review and any attached files.

If you choose “no”, your identity will remain anonymous but your review may still be made public.

Do you want your identity to be public for this peer review? For information about this choice, including consent withdrawal, please see our Privacy Policy.

Reviewer #1: No

Reviewer #2: No

Reviewer #3: No

Reviewer #4: No
