# Supplementary material for: Maternal dietary practices, dietary diversity, and nutrient composition of diets of lactating mothers in Jimma Zone, Southwest Ethiopia
Source: PLoS One. 2021 Jul 12;16(7):e0254259. doi: 10.1371/journal.pone.0254259 (PMC8274933; doi:10.1371/journal.pone.0254259)
Supplement: Attachment — Submitted filename: PONE-D-20-19141R1_Reviewer Report .docx [file pone.0254259.s006.docx]

Reviewer Report

Manuscript ID: PONE-D-20-19141R1

Significant revision were made and the revised manuscript is more appropriate and ready for publication. I have some concerns, which I stated as follows.

1. The two versions of abstract (abstract copied and pasted in the manuscript submission system and abstract in the main manuscript) are not the same.
2. Sample recruitment period in the original manuscript was changed from March to May 2014 to February 2014 (one month back). Why did the authors change on the period of sample recruitment?
3. Tools (DD) in the original was 9 but changed to ten. This has to be clarified.

**Result**

4. Table 5: Model adequacy is marginally significant (p=0.052) suggesting the model does not correctly fit. For which variable does maximum standard error of 0.543 mean?
5. Two important findings need further explanation. The first finding is women who had increased agricultural productivity had better dietary diversity. Another finding is urban women were better in DD than rural women. It may be true to say agriculture activity is more common in rural than urban. Thus, from these statements, one can assume rural women could be better in DD than urban because of increased agricultural productivity. How do authors explain these controversial findings?

**Discussion**:

6. Line 309 (…we have observed that most households add protein rich ingredients…). Nothing was said in methods section to clarify this study also involved observation.
7. Line 338, it is uncommon to write like this (“According to (34) …). Instead, it would be more informative to re-write what was said by another study).

**Strength**:

8. It is not clear what does “an official food analysis methodology” mean? This was not described elsewhere.
