# Supplementary material for: Maternal dietary practices, dietary diversity, and nutrient composition of diets of lactating mothers in Jimma Zone, Southwest Ethiopia
Source: PLoS One. 2021 Jul 12;16(7):e0254259. doi: 10.1371/journal.pone.0254259 (PMC8274933; doi:10.1371/journal.pone.0254259)
Supplement: Attachment — Submitted filename: Response to Reviewers.docx [file pone.0254259.s007.docx]

# Dear editor,

We have accepted the critical inputs of the reviewers with great appreciation as it improves our manuscript very much. We have addressed their queries point by point as follows:

# Journal Requirements:

Please review your reference list to ensure that it is complete and correct. If you have cited papers that have been retracted, please include the rationale for doing so in the manuscript text, or remove these references and replace them with relevant current references. Any changes to the reference list should be mentioned in the rebuttal letter that accompanies your revised manuscript. If you need to cite a retracted article, indicate the article’s retracted status in the References list and also include a citation and full reference for the retraction notice.

[Note: HTML markup is below. Please do not edit.]

**Authors' response:**

- We found out that none of the references are retracted.
- For one reference (23) title of the citation is updated.
- Three references (4, 23 & 37) had changed their internet site, which is updated in the current version.
- For nine references (3, 11, 15, 16, 17, 26, 27, 28 and 38) the internet sites where the documents are available.
- Two new references (30, 31) are added to the reference list.

Reviewers' comments:

5. Review Comments to the Author

# Reviewer #1:

**Reviewer 1 comment 1:**

Need to attend to all the comments from the reviewers eg. the reference to cash crop should rather be substituted by the word coffee as it is confusing

**Authors' response 1:**

The following corrections were made as suggested by the reviewer.

Line 91: “Cash crop” is changed to “coffee”.

Line 190: Inside Table 1, “cash crop” is changed to “coffee”.

Line 225: Inside Table 4, “cash crop” is changed to “coffee”.

Line 304: “cash crop” is deleted.

**Reviewer 1 comment 2:**

Please review the maternal dietary practices paragraph and reword line 119 which is rather deceptive and subject to multiple interpretations.

**Authors' response 2:**

Line 124-126: The sentence was revised as follows:

“Maternal knowledge and practice in use of insecticide-treated net, consumption of two additional meals in a day, consumption of a variety of food groups, daily use of iodized salt and family planning were assessed (17).”

# Reviewer #2:

**Reviewer 2 comment 1:**

I thank the authors for addressing the previous comments.

**Authors' response 1:**

Thank you.

**Reviewer 2 comment 2:**

Abstract: Some minor issues with English remain (e.g. line 41 "anti-nutritional factors contents" the word factors is probably not required;

**Authors' response 2:**

The following corrections were made as suggested by the reviewer:

Line 43: Rewritten as “The content of anti-nutritional factors…”

Line 26: Rewritten as “The content of anti-nutritional factors…”

Line 269: Rewritten as “Table 7 The content of minerals and anti-nutritional factors…”

**Reviewer 2 comment 3:**

line 43 should be "the majority").

**Authors' response 3:**

Line 46: “majority” is changed to “most”.

**Reviewer 2 comment 4:**

Introduction: Appropriate.

**Authors' response 4:**

Ok

**Reviewer 2 comment 5:**

Methods: Line 92, the reference should perhaps be at the end of the sentence.

**Authors' response 5:**

Line 95: The reference is moved to the end of the sentence as suggested by the reviewer.

**Reviewer 2 comment 6:**

Line 95, a brief description of kebele and woreda would be useful to the reader.

**Authors' response 6:**

Line 98: The following phrase is added to describe kebele:

“(the smallest administrative unit of Ethiopia)”

Line 98-99: The following phrase is added to describe woreda:

“(the third-level of the administrative division of Ethiopia - after zones and the regional states)”

**Reviewer 2 comment 7:**

Statistical analysis section: univariate and multivariate should be univariable and multivariable

**Authors' response 7:**

Line 168: “multivariate” is changed to “multivariable” as suggested by the reviewer.

**Reviewer 2 comment 8:**

Ethics section: Line 169-170: This should be "The Ethical Review Board agreed to verbal consent as the study was not an intervention"

**Authors' response 8:**

Line 176-177: Corrected as suggested by the reviewer.

**Reviewer 2 comment 9:**

Line 170, the word "Besides" is not required

**Authors' response 9:**

Line 177: The word “Besides” is deleted as suggested by the reviewer.

**Reviewer 2 comment 10:**

Results: Line 197, either dietary diversity score needs (DDS) after it in order to define the abbreviation in the next line or if already defined DDS should be used rather than "dietary diversity score"

**Authors' response 10:**

Line 204: “dietary diversity score” is changed to “DDS” as it is already defined in the abstract.

**Reviewer 2 comment 11:**

Line 220, For clarity "On multivariable logistic regression analyses" should be "Multivariable logistic regression analyses are.." a full stop is required after Table 5 and then the next sentence becomes "After adjusting..

**Authors' response 11:**

Line 228: Corrected as suggested by the reviewer:

“Multivariable logistic regression analyses are presented in Table 5. After adjusting…”

**Reviewer 2 comment 12:**

Line 221, if Minimum dietary diversity has been defined, please use the abbreviation MDD

**Authors' response 12:**

As minimum dietary diversity for women (MDD-W) is defined in the abstract (Line 27), the abbreviation MDD-W is used in the following instances:

Line 33, Line 36, Line 137, Line 140, Line 215 (inside table 3), Line 229, Line 232, Line 234, Line 237-238, Line 247, Line 248, Line 251

**Reviewer 2 comment 13:**

Discussion: Some minor issues with English remain. e.g. Line 310, "Whereas" is a conjunction and does not start a sentence.

**Authors' response 13:**

Line 341: Deleted “whereas” and the sentence now begins with “Only unleavened…)

**Reviewer 2 comment 14:**

Line 357 "Recall bias. Over and under-reporting of maternal dietary intakes." These are not complete sentences.

**Authors' response 14:**

The incomplete sentences were corrected as follows:

Line 388-389:

“Recall bias might have occurred as information on maternal dietary practices were collected retrospectively.”

Lines 389:

“The 24-hour recall is prone to over and under-reporting of maternal dietary intakes.”

**Reviewer 2 comment 15:**

Strength of study, while an official food analysis methodology is somewhat of a strength, I think this section does need more consideration. The sampling methodology could also be considered as strength as it was quite rigorous.

**Authors' response 15:**

Line 398: As suggested by the reviewer, the following sentence is added as a strength of the study.

“Rigorous sampling methodology was used.”

# Reviewer #3:

**Reviewer 3 comment 1:**

What does "women in Omo-nada were less likely consuming diversified diet than women in Dedo district" imply? Any policy implication for this finding?

**Authors' response 1:**

Line 311-316: The following discussion is added to address reviewer’s comments:

“The variation in dietary diversity between vegetable and cereal producing districts indicates that households tend to consume what they have produced; perishables (vegetables) are more likely to be consumed by the producers than durables (cereals) as they spoil fast if not sold. Therefore, increasing the diversity of farm should be encouraged as one key strategy to improve diet diversity and quality (30).”

**Reviewer 3 comment 2:**

Also your analysis showed a year increase in women's age was associated with higher dietary diversity. However, in the discussion you mentioned other studies have found being young age was positively associated with dietary diversity and this in opposite of your finding.

That discussion was presented wrongly in the previous submission. It has been corrected as follows in the current submission:

Line 302-304: “Regarding the association between maternal age and DDS, a contradicting report has been documented from Nigeria where being young age was positively associated with dietary diversity (24).”

# Reviewer #4:

**Reviewer 4 comment 1:**

3 43-44 This sentence would be much more readable if it said, “ranged between 24.8-65.6 for moisture, 7.6-19.8 for protein,” etc… instead of a long string of numbers followed by a long string of components for the reader to match in order to obtain meaning.

**Authors' response 1:**

Lines 37-40: Corrected as suggested by the reviewer.

**Reviewer 4 comment 2:**

4 46 See above.

**Authors' response 2:**

Lines 41-42: corrected as suggested by the reviewer.

**Reviewer 4 comment 3:**

6 102 Description of the sampling is improved by adding the urban/rural stratification. Was the “Probability Proportional to Size” technique actually used to select the kebeles?

**Authors' response 3:**

Line 101-102: This was a mistake and is corrected in the revised version as follows:

“probability non-proportional to size”

**Reviewer 4 comment 4:**

How does the woreda relate to the district?

**Authors' response 4:**

Line 98: The following text is added to show how woreda is related to district.

“district/woreda”

**Reviewer 4 comment 5:**

8 143-144 Minimum dietary diversity was achieved if a mother consumed five or more food groups per day??

**Authors' response 5:**

Lines 137-138: Corrected as indicated by the reviewer.

**Reviewer 4 comment 6:**

12 199 Consider a different title for this section. Unusual to see insecticide-treated nets and family planning under a heading of dietary practices.

**Authors' response 6:**

Line 192: As advised by the reviewer, the section title is revised to “Maternal practice of essential nutrition actions”.

Line 122: Similar modification is done for the section title.

Line 285: Similar modification is done for the section title.

**Reviewer 4 comment 7:**

13 208 Table 2 includes more than nutrition

**Authors' response 7:**

Line 201-202: The caption is revised as follows:

“Maternal practice of essential nutrition actions during lactation in three districts of Jimma Zone, Southwest Ethiopia”

**Reviewer 4 comment 8:**

14 226 Table 3 now lists 10 food groups, but the title still says 9 food groups

**Authors' response 8:**

That was an error. It is corrected.

**Reviewer 4 comment 9:**

16 236 Is it statistically valid to compare the 554 women who were not currently in school with the four women who were in school. How large a cell size is required for Chi-Square?

**Authors' response 9:**

The reviewer is correct. “The Chi-square test is invalid if we have fewer than 5 observations in a cell”. Therefore, we have deleted, from Table 4, the finding presenting whether the mother is currently attending education or not.

Line 220: deleted “whether the woman is currently attending education or not,”

Line 222: deleted “who are not currently attending education,”

**Reviewer 4 comment 10:**

17 Table 4 See question above.

**Authors' response 10:**

Line 225-226: We have deleted, from Table 4, the finding presenting whether the mother is currently attending education or not.

**Reviewer 4 comment 11:**

17 Table 4 Add names of districts in parentheses here to make Table 5 meaningful.

**Authors' response 11:**

Names of the districts were put in a more meaningful fashion as follows.

Coffee producing (Mana)

Cereal producing (Omo Nada)

Vegetable producing (Dedo)

**Reviewer 4 comment 12:**

17 Table 4 Some ethnicities were merged into other…….Consider if three more are too small for valid chi-square.

**Authors' response 12:**

As suggested by the reviewer, the fourth ethnicity “Tigre” was added to “Others” because it would be too small for a valid chi-square.

**Reviewer 4 comment 13:**

18 251 & 254 “grade of the women”……This should be educational level or educational status…..not grade, because individual grade completed is not shown in Table 4.

**Authors' response 13:**

Line 34, Line 221, Line 223, Line 226 (inside Table 4), Line 230, Line 233, Line 239 (inside table 5): We have changed “grade” with “educational level” for the women as well as their husbands.

**Reviewer 4 comment 14:**

18 Table 5, L. 252 Food production diversity and agricultural production diversity appear but how they are identified has not been presented in previous tables. How is a unit increase defined? Is it an additional type of crop?

**Authors' response 14:**

Line 240-241: At the footnote of Table 5, we have indicated how the production diversity score is generated. Production diversity is a score generated by summing the number of animal and plant source food that the household reported to produce. A unit increase in this context can be defined as an additional type of animal or plant source food produced by the household.

**Reviewer 4 comment 15:**

19 274-277 See first comment

**Authors' response 15:**

Line 255-260: Corrected as suggested by the reviewer.

**Reviewer 4 comment 16:**

20-21 Tables 6 & 7 Clarify if units are /wet_wt or /dry wt somewhere on each of these tables

**Authors' response 16:**

Line 263: The following information is added to the Table 6 caption:

“(dry weight basis)”

Line 269: The following information is added to the Table 7 caption:

“(dry weight basis)”

**Reviewer 4 comment 17:**

22 295-298 Unless I missed something in the methodology, proximate analysis of individual foods was conducted. ..most of the foods eaten by women did not contain…

And, …The overall adequacy is less than 1 for each of the food types shown in Table 8.

**Authors' response 17:**

As indicated by the reviewer, proximate, mineral and anti-nutrient contents analysis of individual foods was conducted.

Line 275-276: “…did not contain...” is changed to “…do not provide the recommended daily allowances for…”

Line 278-279: The following phrase is added to elaborate what MAR<1 means:

“…which indicates the requirements for all the nutrients were not met.”

**Reviewer 4 comment 18:**

22 Table 8 MC is in the footnote but not the table.

**Authors' response 18:**

MC=Moisture content is deleted from the footnote of Table 8 as it happened to be there by mistake.

**Reviewer 4 comment 19:**

Table 8 Use Zn in the heading.

**Authors' response 19:**

Corrected.

**Reviewer 4 comment 20:**

25 351 Confirm the RDA for fiber

**Authors' response 20:**

According to the American Heart Association, the daily value for fiber for women under 50 is 21 to 25 grams per day. The value in Table 6 is revised accordingly.

**Reviewer 4 comment 21:**

27 410 The practice of adding beans to kale sauce is mentioned in the paper, but the food product is still referred to as “kale sauce”. To someone who has knowledge of what % protein typically, would be provided by “kale” alone, this is puzzling. Isn’t the addition of beans critical to the protein concentration being reported?

**Authors' response 21:**

This is a legitimate concern. To avoid the confusion, we renamed the “kale sauce” to “kale-bean sauce” in Tables 6, 7 and 8.

# Reviewer # 5

**Reviewer 5 comment 1:**

Significant revision were made and the revised manuscript is more appropriate and ready for publication. I have some concerns, which I stated as follows.

1. The two versions of abstract (abstract copied and pasted in the manuscript submission system and abstract in the main manuscript) are not the same.

**Authors' response 1:**

This is corrected.

**Reviewer 5 comment 2:**

2. Sample recruitment period in the original manuscript was changed from March to May 2014 to February 2014 (one month back). Why did the authors change on the period of sample recruitment?

**Authors' response 2:**

March to May 2014 is when we collected the data. The preliminary tasks were completed ahead of the data collection.

**Reviewer 5 comment 3:**

3. Tools (DD) in the original was 9 but changed to ten. This has to be clarified.

**Authors' response 3:**

This was suggested by the reviewer during the first revision, and we agreed with the reviewer’s comment. The FAO, FHI 360 (2016) guide for measurement of Minimum Dietary Diversity for Women (MDD-W) is the most applicable technique for the study population.

**Reviewer 5 comment 4:**

**Result**

4. Table 5: Model adequacy is marginally significant (p=0.052) suggesting the model does not correctly fit. For which variable does maximum standard error of 0.543 mean?

**Authors' response 4:**

We expected Hosmer-Lemeshow test to be non-significant (P>0.05) for fitness of the model based on literature. The value we got for Hosmer-Lemeshow test in this analysis was non- significant (P=0.052). Our conclusion therefore is the model is fit.

Large standard error is an indicator of multicollinearity as approximately 95% of the observations should fall within plus/minus 2 standard error of the regression from the regression line. So, we used Standard error of > 2 as an indicator of multicollinearity and checked it for all variables in the multivariable regression output. However, all variables had low standard error with the maximum standard error being. 0.543 (for educational status of the husband) showing the absence of multicollinearity.

**Reviewer 5 comment 5:**

5. Two important findings need further explanation. The first finding is women who had increased agricultural productivity had better dietary diversity. Another finding is urban women were better in DD than rural women. It may be true to say agriculture activity is more common in rural than urban. Thus, from these statements, one can assume rural women could be better in DD than urban because of increased agricultural productivity. How do authors explain these controversial findings?

**Authors' response 5:**

Line 319-327: The following explanation is added to explain the urban-rural disparity in DD.

“Considering the fact that rural areas are hubs for agricultural production, one can assume rural women could be better in dietary diversity than urban counterparts. However, production diversification may not always mean dietary diversity. For example, a study in rural Nigeria found out that production diversification has no statistically significant effect on the dietary diversity of households (30). Other researchers also argue that despite theoretical basis for the correlation between production diversity and dietary diversity, “there is a need for a deeper empirical understanding of how, under what circumstances, and through what pathways own-production of nutritious foods improves diets” (31).”

The following new references were added to the reference list:

Line 525-528:

“Ayenew HY, Biadigilign S, Schickramm L, Abate-Kassa G, Sauer J. Production diversification, dietary diversity and consumption seasonality: Panel data evidence from Nigeria. BMC Public Health [Internet]. 2018 Aug 8 [cited 2021 Jun 15];18(1):1–9. Available from: https://doi.org/10.1186/s12889-018-5887-6”

Line 529-531:

“Aberman N-L, Roopnaraine T. To sell or consume? Gendered household decision-making on crop production, consumption, and sale in Malawi. Food Secur [Internet]. 2020;12(2):433–47. Available from: https://doi.org/10.1007/s12571-020-01021-2”

**Reviewer 5 comment 6:**

**Discussion**:

6. Line 309 (…we have observed that most households add protein rich ingredients…). Nothing was said in methods section to clarify this study also involved observation.

**Authors' response 6:**

We didn’t do observational study, but we have collected food samples from several households. We have revised the sentence to this context.

Line 337: “…our survey…” is changed to “…collection of food samples,…”

**Reviewer 5 comment 7:**

7. Line 338, it is uncommon to write like this (“According to (34) …). Instead, it would be more informative to re-write what was said by another study).

**Authors' response 7:**

Line 367-368: Corrected as suggested by the reviewer:

“The ideal cut-off for nutrient adequacy should be 1, which would mean that all the nutrients were consumed in a sufficient amount (36).”

**Reviewer 5 comment 8:**

**Strength**:

8. It is not clear what does “an official food analysis methodology” mean? This was not described elsewhere.

**Authors' response 8:**

Line 147: “standard methods” was changed to “official methods”

Thank you
